# Combined Targeted DNA Sequencing in Non-Small Cell Lung Cancer (NSCLC) Using UNCseq and NGScopy, and RNA Sequencing Using UNCqeR for the Detection of Genetic Aberrations in NSCLC

**DOI:** 10.1371/journal.pone.0129280

**Published:** 2015-06-15

**Authors:** Xiaobei Zhao, Anyou Wang, Vonn Walter, Nirali M. Patel, David A. Eberhard, Michele C. Hayward, Ashley H. Salazar, Heejoon Jo, Matthew G. Soloway, Matthew D. Wilkerson, Joel S. Parker, Xiaoying Yin, Guosheng Zhang, Marni B. Siegel, Gary B. Rosson, H. Shelton Earp, Norman E. Sharpless, Margaret L. Gulley, Karen E. Weck, D. Neil Hayes, Stergios J. Moschos

**Affiliations:** 1 Lineberger Comprehensive Cancer Center, the University of North Carolina at Chapel Hill, Chapel Hill, NC, 27599, United States of America; 2 Department of Pathology and Laboratory Medicine, the University of North Carolina at Chapel Hill, Chapel Hill, NC, 27599, United States of America; 3 The University of North Carolina at Chapel Hill School of Medicine, Chapel Hill, NC, 27599, United States of America; 4 Department of Genetics, the University of North Carolina at Chapel Hill, Chapel Hill, NC, 27599, United States of America; 5 Department of Medicine, the University of North Carolina at Chapel Hill, Chapel Hill, NC, 27599, United States of America; 6 Department of Pharmacology, the University of North Carolina at Chapel Hill, Chapel Hill, NC, 27599, United States of America; 7 Department of Biostatistics, the University of North Carolina at Chapel Hill, Chapel Hill, NC, 27599, United States of America; University of Torino, ITALY

## Abstract

The recent FDA approval of the MiSeqDx platform provides a unique opportunity to develop targeted next generation sequencing (NGS) panels for human disease, including cancer. We have developed a scalable, targeted panel-based assay termed UNCseq, which involves a NGS panel of over 200 cancer-associated genes and a standardized downstream bioinformatics pipeline for detection of single nucleotide variations (SNV) as well as small insertions and deletions (indel). In addition, we developed a novel algorithm, *NGScopy*, designed for samples with sparse sequencing coverage to detect large-scale copy number variations (CNV), similar to human SNP Array 6.0 as well as small-scale intragenic CNV. Overall, we applied this assay to 100 snap-frozen lung cancer specimens lacking same-patient germline DNA (07–0120 tissue cohort) and validated our results against Sanger sequencing, SNP Array, and our recently published integrated DNA-seq/RNA-seq assay, UNCqeR, where RNA-seq of same-patient tumor specimens confirmed SNV detected by DNA-seq, if RNA-seq coverage depth was adequate. In addition, we applied the UNCseq assay on an independent lung cancer tumor tissue collection with available same-patient germline DNA (11–1115 tissue cohort) and confirmed mutations using assays performed in a CLIA-certified laboratory. We conclude that UNCseq can identify SNV, indel, and CNV in tumor specimens lacking germline DNA in a cost-efficient fashion.

## Introduction

Use of next generation sequencing (NGS) for large-scale analysis of DNA sequence alterations in human tissue, which may be related to etiopathogenesis of disease, is not only useful in basic science studies, but is now an established laboratory technique used in clinical medicine, in particular for the care, of patients with distant metastatic cancer (reviewed in [1]). Implementation of NGS as a standard clinical laboratory test is the next logical step following FDA approval of several first generation sequencing-based companion diagnostic tests over the last decade that refine the use of targeted gene variants for managing distinct cancer subtypes. In line with FDA approval of the MiSeqDx platform in November 2013, targeted panel sequencing (TPS) is the next step towards implementing affordable, small-scale, NGS-based laboratory diagnostics [2].

FDA approval of a generic platform for NGS has encouraged individual laboratories to address the inherent challenges associated with development of such tests. These challenges involve fiscal matters, issues in methodology and optimal bioinformatics pipelines that offer a reasonable compromise between technical sophistication and time efficiency. Since various laboratories address such matters differently, dissemination of information regarding methods and performance characteristics of a particular NGS-based laboratory assay is a basis for discussion and evaluation of strengths and weaknesses by the scientific community.

**Fig 1:**
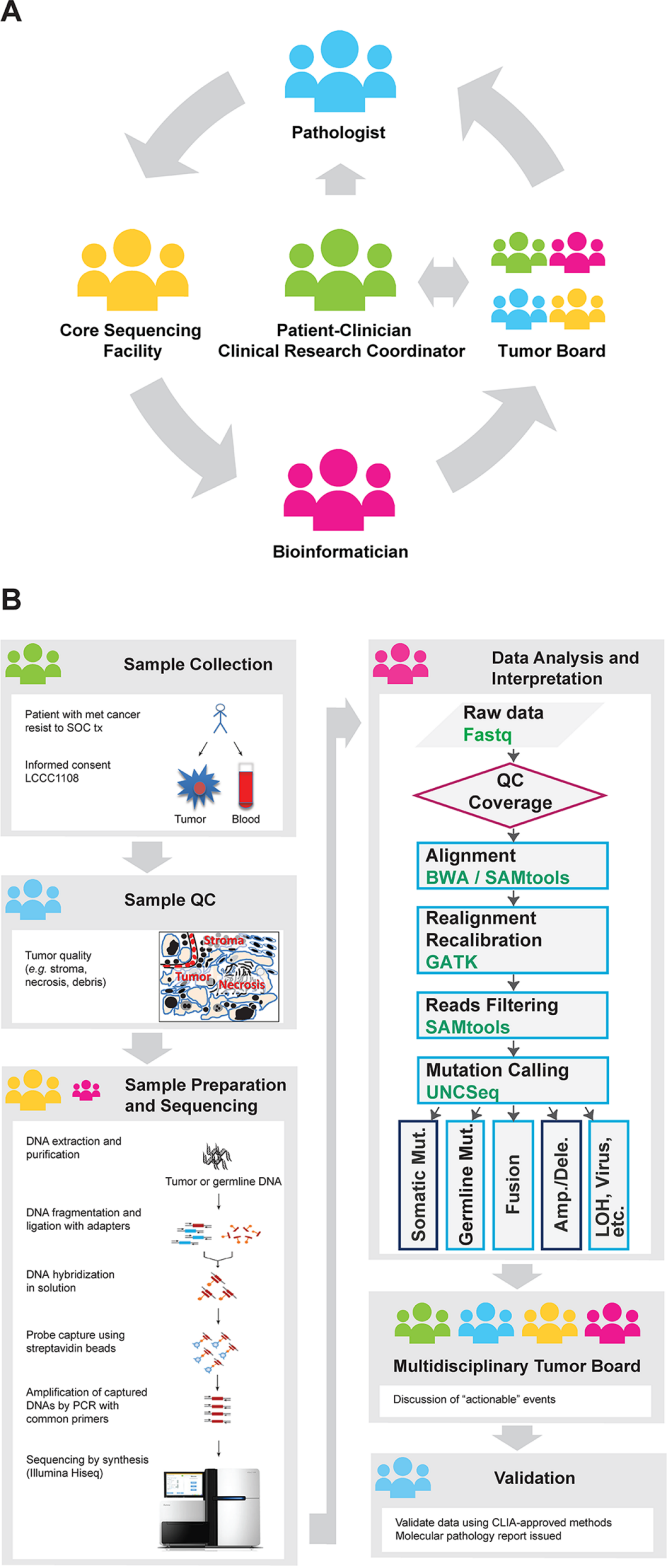
The UNCseq project. **(A)** The UNCseq project is an initiative that involves clinicians and patients interested to participate in a non-therapeutic clinical trial conducted through the Lineberger Comprehensive Cancer Center (IRB-approved protocol 11–1115), as well as a multidisciplinary team that involves clinical and research faculty (medical oncologists, pathologists, bioinformaticians, and molecular biologists) who generate, critically assess, and discuss NGS data in relation to patients’ clinical history and review previously identified genetic aberrations to determine which are potentially clinically actionable and targeted for downstream validation using validated methods in a CLIA-certified laboratory. **(B)** Following consent to 11–1115, tumor tissues and peripheral blood are collected from cancer patients. Hematoxylin and eosin (H&E)-stained representative tissue sections from tumor samples (SF or FFPE) are assessed by a certified pathologist for the percentage of viable tumor/stroma content and presence/absence of necrosis (sample QC). Extracted DNA from tumor samples is processed through various steps (fragmentation, DNA library preparation, in-solution capture of DNA fragments of interest, small-scale amplification of captured DNA fragments) prior to Illumina NGS. Data generated are discussed in a multidisciplinary Molecular Tumor Board meeting. Following validation in a CLIA-certified laboratory, these genetic aberrations are reported in patients’ personal electronic medical records.

In line with this, an increasing number of reports of NGS-based laboratory methods to analyze clinical tumor specimens by different laboratories for clinical decision were recently published [1, 3–8]. At the University of North Carolina at Chapel Hill (UNC-CH), we developed a scalable NGS assay (UNCseq) that involves TPS of DNA obtained from tumor and matched non-malignant specimens for a gene panel (ClinSeq) of over 200 cancer-associated genes that were selected and updated quarterly by UNC Committee for the Communication of Genetic Research Results (CCGR). In addition, UNCseq developed a standardized downstream bioinformatics pipeline, which is currently being used to order confirmatory tests for reporting clinically ‘actionable’ genetic events to the treating physician under an Institutional Review Board (IRB)-approved study (Fig 1). In this report, we test our ability to successfully perform Illumina HiSeq 2000 sequencing upon DNA extracted from tumor specimens from patients with lung cancer, in particular, the non-small cell lung cancer (NSCLC) subtype. In addition, we summarize our experience in sample acquisition, pathologist-vetted tumor diagnosis, DNA extraction, NGS, and analytical validation of genetic results. Finally, we provide our experience of applying this NGS-based assay in reporting somatic mutations from ‘real-world’ samples—both snap-frozen (SF) and formalin-fixed and paraffin-embedded (FFPE)—for diagnostic purposes with validation of results in a CLIA-certified laboratory. We confirmed that TPS in a well annotated lung cancer cohort is not only a more sensitive method than Sanger sequencing in SNV detection, but also more specific to identify genetic aberrations in known cancer-related genes with important prognostic and treatment implications. By performing deep sequencing of cDNA prepared from RNA (RNA-seq) in a subset of these samples, we also confirmed several SNV detected by the sequencing of DNA (DNA-seq), depending on the coverage depth by RNA-seq and the mutant allele frequency (MAF) by DNA-seq. Given the fact that matched normal DNA may not always be available, we provide systematic comparison of SNV calling algorithms using matched germline versus pooled normal DNA, and versus mere tumor genotyping in a subset of those specimens. Finally, we present a new algorithm, *NGScopy* (http://www.bioconductor.org/packages/release/bioc/html/NGScopy.html), to detect genome-wide CNV using TPS data. We conclude that our NGS-based laboratory assay is sensitive, yet specific, cost-effective, robust, and standardized, and facilitates downstream bioinformatics analysis to assess SNV, indel, and CNV in a time-efficient and clinically impactful manner.

## Materials and Methods

### Patients, Tumors, and Histopathologic Assessment

Under the IRB and Office of Human Research Ethics, The University of North Carolina at Chapel Hill (UNC-CH), approved protocol 07–0120, patients who underwent standard of care (SOC) surgery for primary lung cancer were identified, followed by retrieval of SF, banked tumor tissues (07–0120 tumor tissue cohort; n = 100). A separate cohort of patients with lung cancer who had become refractory to standard systemic treatments was consented under the IRB and Office of Human Ethics, UNC-CH approved protocol 11–1115 (11–1115 tumor tissue cohort; n = 24). Written informed consent from the subject patients was obtained for the use of these samples in research. The 11–1115 protocol allows for TPS of SF or archived FFPE tumor tissues and same-patient germline DNA to identify genetic aberrations of prognostic or therapeutic significance using the UNCseq assay. Genetic aberrations that are identified under the UNCseq assay and have potential clinical significance are then subjected to validation in a CLIA-certified laboratory only for the 11–1115 tumor tissue cohort (Fig 1). In addition, tumor content for each specimen of both cohorts was estimated based on routine microscopic analysis of representative hematoxylin and eosin (H&E)-stained sections from adjacent tumor by a pathologist (XY) who was blind to patient history.

### DNA Library Preparation and Capture

5 μm-thick tissue sections were prepared from SF or FFPE tumor tissues. DNA was isolated using the Gentra Puregene Tissue Kit (QIAGEN, Valencia, CA). 3 μg of DNA was then sheared for 60–90 seconds using the Covaris ultrasonicator instrument (E220) following manufacturer instructions (Covaris Inc., Woburn, MA). Non strand-specific DNA library preparation was performed using an Agilent SureSelectXT Reagent kit with custom target enrichment following manufacturer’s recommendations (Agilent Technologies Inc., Santa Clara, CA). DNA was then subjected to repair, end-polishing (blunt-end or A-overhang), and ligation of custom, single-end adapters. Libraries were then captured with biotinylated RNA baits designed by Agilent Technologies to separate exonic sequences for a consensus list of genes associated with cancer. More specifically, the genes were selected by UNC CCGR from publications and from the periodically updated Catalogue of Somatic Mutations in Cancer (COSMIC) database [9], based on the frequency of mutation(s) in solid tumors, their potential role in oncogenic pathways, and their potential relevance of antitumor response to small molecule inhibitors. This gene list is updated on a quarterly basis by the UNC CCGR according to new research and medical findings [UNCseq ClinSeq versions 4, 5 (07–0120 tumor tissue cohort), and version 7 (11–1115 tumor tissue cohort); S1 Table]. A set of genomic region targets that cover all exons for each gene was developed based on the University of California at Santa Cruz (UCSC) Known Gene database [10, 11]. Regions of the targeted exons for capture were extended to include 250 base pairs (bp) of flanking sequences in intronic regions to comprehensively cover targeted genes. These genomic locations provided the basis for the design of 120 nucleotide (nt) biotinylated capture oligos for Agilent SureSelect capture using the Agilent eArray web portal (https://earray.chem.agilent.com/suredesign/). Each kit targeted 3,379 (ClinSeq v4), 3,323 (ClinSeq v5), or 5,997 (ClinSeq v7) regions spanning 2,231,841-bp for a total of 228 genes (ClinSeq v4), 3,451,622-bp for a total of 184 genes (ClinSeq v5), and 2,820,216-bp for a total of 248 genes (ClinSeq v7) (S1 Table). Capture of barcoded-and-pooled or unpooled libraries was processed by the Agilent SureSelect Protocol.

Prior to submission for NGS, DNA libraries were subjected to a three-step quality control protocol. DNA concentration was measured using a Qubit 2.0 fluorometer (Life Technologies, Grand Island, NY), DNA quality was assessed using Agilent’s 2100 Bioanalyzer high sensitivity DNA assay, and DNA size was determined by the Experion automated electrophoresis system (BioRad, Hercules, CA). A normalized molarity for each library was then calculated based on DNA size and concentration. Libraries were pooled to include 2–8 samples per sequencing lane. Each pool was diluted into 5.5 pM, as per the Illumina cBot Cluster Generation step. Clusters were then generated using TruSeq SR Cluster Kit v.2 and were loaded into the HiSeq 2000 sequencer (Illumina Inc., San Diego, CA). Sequencing by synthesis [12] was performed using standard single-indexed libraries on either single-read (07–0120) or paired-end (11–1115) flow cells with 100 cycles (ClinSeq 1 x 100-bp or 2 x 100-bp, respectively) and an index read (‘barcode’) consisting of 7 cycles of sequencing using the Illumina TruSeq SBS v.3 chemistry. S2 Table summarizes key differences in sample processing and sequencing between the 07–0120 and 11–1115 tumor tissue cohorts.

### DNA NGS Data Analysis Pipeline

#### Preprocessing, Pre-filtering, Alignment, and Filtering

The data analysis pipeline is shown in Fig 1. No strand-bias was considered in any of the pre-processing steps. Raw sequence reads were analyzed using the CASAVA v.1.8 package (Illumina) to generate barcoded reads and were reported as FASTQ files [13]. If applicable, reads were then subjected to quality-filtering and adapter-stripping using the FASTX-Toolkit (http://hannonlab.cshl.edu/fastx_toolkit/index.html). The Phred quality score of base calling (CallQ) of each nucleotide in a read was then examined to determine whether to trim the read at the ends when a number of continuous nucleotides average per-base CallQ ≤ 20, or ≤ 99% accuracy. The raw sequence reads in FASTQ files were then aligned to the Genome Reference Consortium human genome, build 37 (GRCh37; http://www.ncbi.nlm.nih.gov/assembly/GCF_000001405.13/), using either the Burrows-Wheeler Aligner [14] (BWA 0.6.2) for the 07–0120 cohort or the BWA-MEM (version 0.7.4) for 11–1115 cohort. Reads were then sorted and indexed using SAMtools (0.1.19-44428cd) [15]. Local realignment and base quality score recalibration were performed using either the Genome Analysis Toolkit (GATK 2.6) and the GATK resource bundle (2.5) [16] in 07–0120 cohort or the ABRA (0.46) [17] in 11–1115 cohort. Default parameter settings were used with tools above. Mapped reads were further filtered by mapping quality before downstream analysis. Filtering was performed by imposing a minimum Phred quality score of read mapping (MapQ). Reads with low mapping quality (MapQ < 5, i.e. < 70% accuracy) were removed. Median and approximate 95% confidence interval (approx. 95% CI) were calculated for on-target reads for each tumor cohort and for each ClinSeq caption version. The median of the per-sample median RPKM (reads per region kilobase per million targeted reads mapped) [18] was used to describe the average reads per region.

#### Quality Control

Depth, breadth of coverage, and on-target rate were computed according to definitions outlined **in** S1 Text.

#### Variant Calling

Due to the retrospective specimen collection of the 07–0120 tumor tissue cohort, DNA from same-patient normal tissues (e.g., peripheral blood) was not available to extract germline DNA. For control DNA, we instead sequenced and pooled DNA that was extracted from 8 normal tissues (6 liver and 2 uterus from a total of 4 patients) under similar conditions and treatment protocols applied to those for DNA-seq of tumor samples. Genetic variants were called by deepSNV [19]. SNV calls from our assay were further refined using the prior knowledge from a highly curated list of 41 genes with 279 SNV and 91 indel positions that have been used by the OncoMap system (version 4; an expert curated source that we call ‘conservative’ list) [20] and the COSMIC database (version 66) with annotation in lung cancer only. We call the COSMIC list ‘less-conservative’ as it consists of 18,722 genes with 250,741 SNV and 4,949 indel positions; 265 out of these 18,722 genes that have no genomic coordinate information of the variants were excluded [9]. Of note, all genes and SNV/indel positions of the OncoMap system are all annotated in the ‘less-conservative’ list, and therefore the latter is also referred to as the OncoMap plus COSMIC system.

For variant calling on the 07–0120 tumor cohort, we defined significant SNV by filtering each of the mutation calls using the ‘deepSNV’ package with Bonferroni-adjusted *p*-value ≤ 0.001, MAF ≥ 0.005, mutant allele read count (MAC) in tumor ≥ 5, and the logarithmically transformed (log_2_) odds ratio (OR)[21] of MAC of each individual tumor sample versus the pool of normal samples ≥ 4. In other words, the odds of calling a SNV in each individual tumor sample were ≥ 16 (i.e., 2^4^) times higher compared to the pooled normal. We selected this MAF threshold because it was at least two times higher than the previously reported sequencing error of approximately 0.001–0.002 [22]. Regarding the MAC threshold, we arbitrarily set it to 5, which is more strict than MAC > 2 that was previously reported [23]. Filtered SNVs were annotated by ANOVAR (2014.07.14). To improve confidence in calling unmatched tumors, SNV were further refined using the ‘conservative’ list [24] as well as the ‘less-conservative’ list.

Based on gene-wise aggregation of the significant SNV identified above, each individual gene was then tested under the null hypothesis that the mutation rate across the gene is in accordance with the background mutation rate, to obtain a *p*-value using a conventional binomial probability model [25] to adjust mutation rates for gene length. Finally, the SMG were reported using the significant level of mutated genes for all tested genes with false discovery rate (FDR) ≤ 0.05. Indel were called by VarScan (2.3.6) with default setting.

Variant calling of the 11–1115 tumor tissue samples was performed by the updated version of the UNCseq pipeline (August 2014). More specifically, we used the strelka somatic variant caller (2013) with default settings [26] to detect both SNV and indel with quality scores of at least 30 for both, ANOVAR (version 2014.07.14) to annotate detected variants, and SAMtools/BCFtools (version 0.1.19-44428cd) for normal-free variant calling. To establish a ‘contemporary pooled’ normal DNA for this tumor tissue cohort, we first generated a ‘leave-one-out’ pooled DNA consisting of all sequenced reads from the available germline DNA of the 11–1115 cohort, excluding the matched germline DNA for the particular sample. In other words, for a given i-th tumor sample, the pooled normal consisted of 23 normal samples from patients 1, 2, …, i-1, i+1, …,n (n = 24). As a second step, we subsampled the total reads from the pooled normal DNA to reduce the computational time, and generated a comparable size of contemporary library for optimal statistical analysis. The S2 Table summarizes key differences in bioinformatics analysis between the 07–0120 and 11–1115 tumor tissue cohorts.

#### Detection of Copy Number Variations

We calculated chromosome-level copy number variations (CNV) in the 07–0120 tumor tissue cohort using the read depth. Due to the inherently heterogeneous, interrupted coverage of the genome by TPS, we employed a ‘restriction-imposed,’ flexible windowing algorithm to ensure a balanced number of reads per window across the entire genome in the R/Bioconductor package *NGScopy* (1.0.0). To enable detection of copy number in both targeted and off-targeted areas of the genome, which usually have high and low coverage depth, respectively, off-target reads (‘background reads’) were used in addition to on-target. Two criteria defined such a flexible window. First, to ensure even variance as well as adequate number of reads per window, the read depth per window in the pooled normal control sample was no less than 20x per sample. Second, its minimal window size was kept within a range determined by coverage characteristics, as in genomic regions with high-read density, the use of small window sizes leads to a ‘sawtooth,’ undersmoothened signal. For this study, the minimum window size used was 20 Kbp. Library size-normalized reads per window for both pooled normal control and each tumor sample were counted to compute the tumor/normal log_2_ copy number ratio (CNR) as the relative copy number. To account for copy number neutrality, we normalized our data per tumor sample by centering the median of the relative copy numbers to zero across the entire genome. Direct visualization was used to assess structural variations across the genome. Finally, segmentation was performed by a heterogeneous hidden Markov model, termed BioHMM [27], which was adapted for NGS data.

To calculate gene-level CNV in the 07–0120 tumor tissue cohort, we used the depth of gene exon-specific sequenced reads with 1-bp resolution. We estimated the relative copy number, similarly as above, by computing the log_2_ ratio of the per-base read depth of the tumor versus the pooled normal control.

#### Validation of DNA NGS Data by RNA Sequencing

Agilent strand-specific RNA with capture was performed for preparation. RNA sequencing (RNA-seq) whole-transcriptome analysis in a subset of tumor samples from the 07–0120 tumor tissue cohort was performed on Illumina GAII as previously described [28, 29]. The full 76-bp, single-end reads were first aligned to the human reference genome (hg19) by MapSplice [30]. SNV called by DNA-seq were subsequently validated by analysis of RNA-seq data using two independent mutation calling algorithms: the SAMtools (mpileup command)/BCFtools [15] and our recently published RNAseq-specific mutation calling method, UNCeqR [31].

#### DNA Non-NGS Assays

For the 07–0120 tumor tissue cohort, we have previously performed Sanger sequencing using a DNA analyzer (ABI 3730xl, Applied Biosystems, Foster City, CA) for mutation detection of selected exons of the *KRAS* gene as well as selected exons of the genes *BRAF*, *CDKN2A*, *EGFR*, *STK11*, and *TP53*. In addition, samples from the 07–0120 cohort were subjected to analysis using the Genome-Wide Human SNP Array 6.0 microarray (Affymetrix, Santa Clara, CA) for detecting CNV in a subset of our lung cancer samples [32]. SNP array analysis for CNV was performed using the open source R package aroma.affymetrix version 2.5.0 (http://cran.r-project.org/web/packages/aroma.affymetrix) and DNACopy version 1.30.0 (http://www.bioconductor.org/packages/release/bioc/html/DNAcopy.html) for data processing and CNV analysis, respectively.

#### Confidence Interval for a Median

Confidence interval (CI) for a median was calculated as previously described [33].

## Results

### Clinicopathologic Characteristics of the 07–0120 and 11–1115 Patient Samples

Tumor tissues from 100 and 24 patients with primary lung cancer were included in the analysis for the 07–0120 and 11–1115 tumor tissue cohorts, respectively. Clinicopathologic characteristics for each cohort are shown in Table 1. Targeted panel capture using ClinSeq versions 4 and 5 were performed in 64 and 36 of the 07–0120 SF samples, respectively, and ClinSeq version 7 was applied to all 24 tumor samples from the 11–1115 tumor tissue cohort. Pooled normal DNA was available for analysis of the 07–0120 tumor cohort, whereas matched germline DNA was available for the 11–1115 tumor cohort. S1 Table shows the list of genes whose exons were sequenced as part of ClinSeq versions 4, 5, and 7.

**Table 1:** Clinicopathologic characteristics of lung cancer specimens for the 07–0120 and 11–1115 tumor tissue cohorts.

**Age at Diagnosis** (Median, Range)	66 (41–82)	61 (41–77)	
**Sex**			
** **Male	55	9	
** **Female	45	15	
**Race**			
** **Caucasian	82	17	
** **African-American	17	3	
** **Asian-American	0	1	
** **Indian-American	0	1	
** **Unknown	1	2	
** Smoking Status**			
** **Current/Former Smoker	74	20	
** **(pack-years; mean, range)	52 (2–141)	32 (0.075–112.5)	
** **Never/ight	7 4		
** **Unknown	19 0		
**Tumor Histology**			
** **Squamous Cell Carcinoma	31	4	
** **Adenocarcinoma or Bronchoalveolar	50	18	
** **Large Cell Carcinoma	7	0	
** **Adenosquamous Carcinoma	7	0	
** **Carcinoid	4	0	
** **Small Cell Carcinoma	1	0	
** **Inconclusive	0	2	
**Tumor Grade**			
** **1	38	1	
** **2	53	9	
** **3	4	8	
** **Unknown	5	6	
**Stage** (AJCC 2007)			
** **I	** **59	2	
** **II	** **27	4	
** **IIIA	8	2	
** **IIIB	0	2	
** **IV	3	14	
** **Unknown	3	0	
**% Tumor in H&E** (Mean, Range)	78 (10–100)	50 (15–90)	
**Mutations by Sanger Sequencing (07–0120 tumor cohort only)**	**No Call**	**No Mutation**	**Mutation Identified**
** ** *KRAS*	0	91	9
** ** *TP53*	12	58	30
** ** *EGFR*	30	64	6
** ** *STK11/LKB1*	6	88	6

### Bioinformatics Analysis of the 07–0120 Patient Samples

We obtained a total of 2,100,991,292 reads from all 64 samples that were sequenced using the ClinSeq version 4, and 591,549,582 reads from all 36 samples that were sequenced using the ClinSeq version 5. All samples have passed quality control using the FASTX-Toolkit. 93.96 ± 0.85% of these reads were uniquely mapped to the reference genome with MapQ ≥ 5, i.e. 1,985,916,272 (94.5%) and 551,493,714 (93.2%) for ClinSeq 4 and 5, respectively. The median number of uniquely mapped (mapQ ≥ 5) reads per sample was 18,171,425 (approx. 95CI 16,442,697–27,015,601) and 14,350,546 (approx. 95CI, 13,786,985–15,363,758) for samples sequenced in ClinSeq versions 4 and 5, respectively. We were able to retrieve 71.6% (median; approx. 95CI, 70.9%-72.5%) and 30.6% (median; approx. 95CI, 29.9–31.4%) on-target bases with our targeted panel capture strategy for ClinSeq version 4 and 5, respectively. The switch from ClinSeq version 4 to 5 was associated with several changes, including ad hoc design of primers by the investigators, as opposed to the vendor (Agilent), as well as new genomic regions of interest whose capture efficiency and ability to readily sequence were questionable. The median of the per-sample median RPKM was 452 (approx. 95CI, 448–458) and 446 (approx. 95CI, 440–454) for samples sequenced using the ClinSeq version 4 and 5, respectively. SNV/indel analysis was restricted to the shared DNA regions for ClinSeq versions 4 and 5, 1,190,667 bases per sample, or 168 genes, for comparison among samples. For copy number analysis, the entire genome was considered, either on-target or not.

A common strategy to overcome the intrinsic high error rate of NGS instruments and to ensure the adequate coverage of both alleles for each variant site or the existence of multiple clones is to ideally sequence individual genomes to 20-30x coverage depth [34]. Such coverage depth is sufficient for a normal tissue, a genetically homogeneous cancer tissue, such as cancer cell lines, or tumor tissue with minimal stromal ‘contamination,’ but not for tumor tissues with variable degree of cellular and/or molecular heterogeneity (i.e., subclones of varying genotype) (Fig 1). A recent study showed that a 30x coverage depth was sufficient for an approximate 90% sensitivity to call mutations at allele fractions of ≥ 0.2 [35]. For the latter cases, a minimum of 50x coverage depth is commonly used to call single nucleotide or other genetic variants.

**Fig 2:**
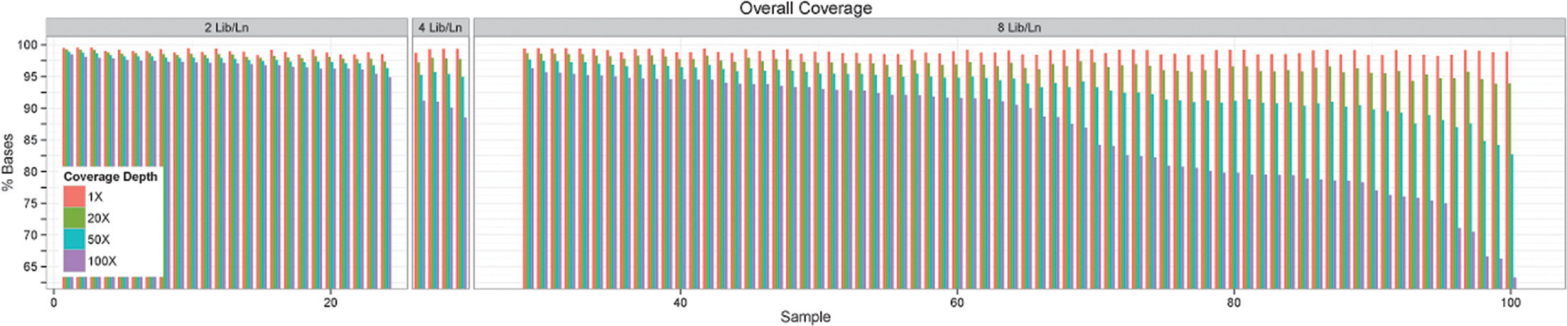
Depth and Breadth of On-Target Coverage of the 100 Lung Cancer Samples. Shown for each tumor specimen is the percentage of targeted bases covered at given coverage depth (1x, 20x, 50x, 100x) and sequenced under different lane settings in the HiSeq 2000 instrument (2, 4, and 8 DNA libraries per lane, Lib/Ln).

To establish the optimal balance between cost and coverage depth for our TPS strategy, we sequenced 2 (n = 24 samples), 4 (n = 4), or 8 samples (n = 72) per flowcell lane. As shown in Fig 2, a target overall coverage depth of 50x was reached when up to 8 samples per lane were loaded. The mean percentages of on-target bases having no less than 50x coverage depth for 2, 4, and 8 samples per lane are 98%, 95%, 93%, respectively; and 97%, 92%, 86%, respectively, for no less than 100x depth. We conclude that 8 samples per lane provide sufficient cost- and time-effective coverage (50x) under our TPS strategy.

### Comparison in SNV Calling Between NGS and Sanger Sequencing in the 07–0120 Patient Samples

**Fig 3:**
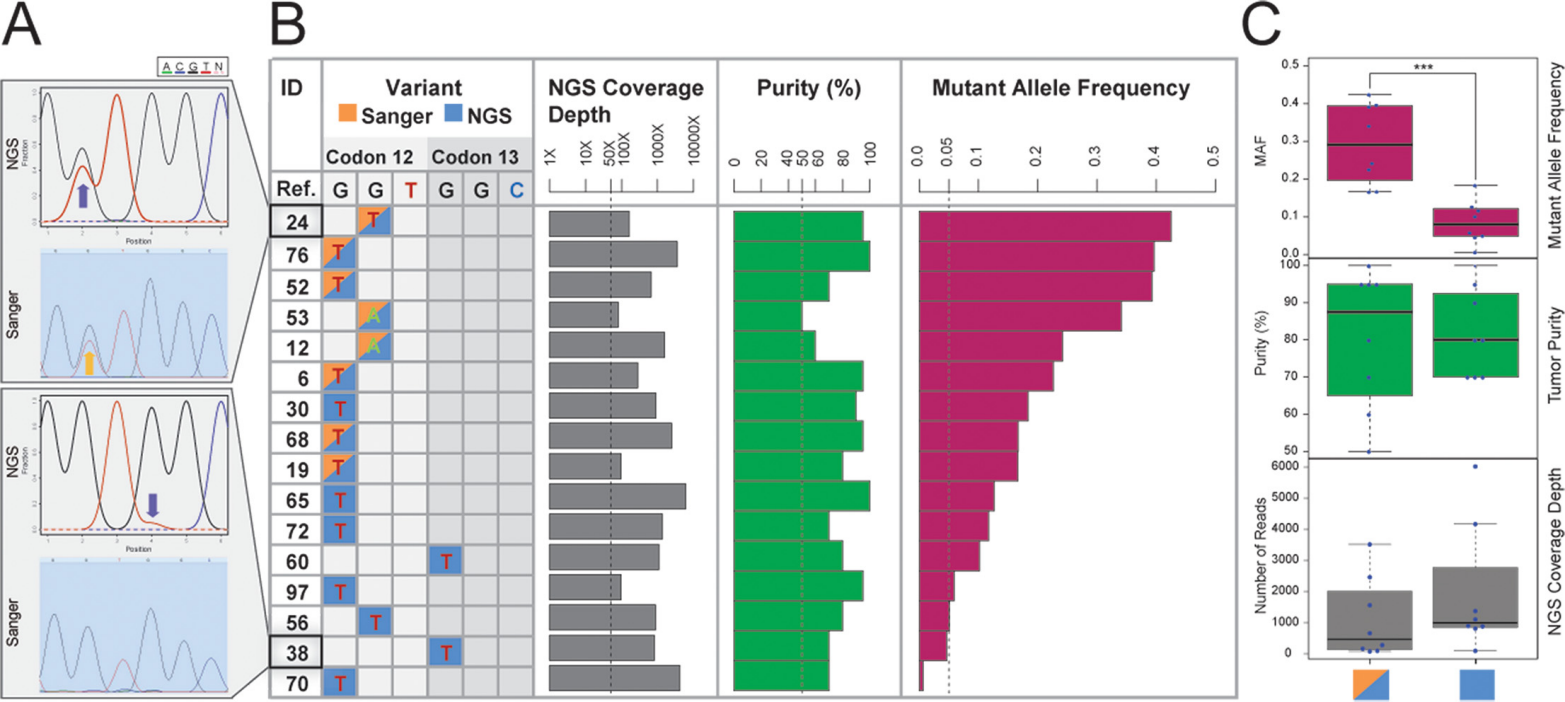
SNV Calling of *KRAS* Hotspots Using First- and Next-Generation Sequencing. **(A)** Sequencing chromatograms (Finch TV trace viewer v1.4.0) obtained from two tumor tissue examples showing concordance (sample 24) or discordance (sample 38) in *KRAS* SNV calling. **(B)** SNV calling at hot-spot loci in *KRAS* codon 12 and 13 for all 16 tumors using either of the two sequencing strategies. Calls by Sanger and NGS are colored in orange and blue, respectively. Calls by both platforms are colored in half orange and half blue. NGS coverage depth, purity, and MAF are also shown. **(C)** Boxplots of MAF, tumor purity, and coverage depth between discordant and concordant SNV calls are shown (*p*-value = 0.0006, two-sided Wilcoxon test).

To assess whether NGS is at least as sensitive as Sanger sequencing in SNV calling for known mutation hotspots, we compared results for detection of *KRAS* hot-spot SNV between the two sequencing platforms. We selected *KRAS* for this investigation because it bears indisputable hotspot somatic SNV for lung cancer in codons 12 and 13, which have been previously well identified [36, 37]. As shown in Fig 3, panels A and B, using our NGS pipeline, we detected all 8 hotspot SNV identified by Sanger sequencing. Furthermore, 8 additional hotspot SNV not identified by Sanger sequencing were also called by our NGS pipeline. As shown in Fig 3, panel C, neither low NGS coverage nor low tumor purity was different between the 8 agreed and the 8 discrepant cases by NGS and Sanger sequencing (*p*-value > 0.1, two-sided Wilcoxon test). Compared to Sanger sequencing, NGS was able to detect the *KRAS* mutant alleles with significantly lower MAF (*p*-value = 0.0006, two-sided Wilcoxon test; Fig 3, panel C). Interestingly, the MAF of 4 discrepant cases (ID: 30, 65, 72, 60) are below but close to 0.20, implying that Sander sequencing is less sensitive to detect SNV with MAF ≤ 0.20, in accordance with previous reports [38]. The MAF of the other 4 discrepant cases (ID: 97,56,38,70) are close to 0.05 or below, indicating NGS was able to capture SNV with very low MAF.

To assess the sensitivity of our NGS SNV calling algorithm, we focused on the first coding exon of *KRAS (RefGene ID*: *NM_033360)*. This 111-bp DNA region (*chr12*:*25*,*398*,*208–25*,*398*,*318*) contains the 6-bp positions corresponding to the hotspot sites in codons 12 and 13 (*chr12*:*25*,*398*,*280–25*,*398*,*285*). Of the remaining 105 bp, there are 52-bp positions with variants annotated by OncoMap plus COSMIC system or dbSNP, and 53-bp positions without variants annotated by either OncoMap plus COSMIC system or dbSNP [39]. Using our SNV calling algorithm, we detected all 9 SNV (8 hotspot SNV as reported above plus 1 SNV at *chr12*:*25398262* of Sample ID: 98) that were independently detected by Sanger sequencing, indicating that NGS is not less sensitive in detecting SNV compared to Sanger sequencing. To evaluate possible false positives, we assume that positive calls falling in the 53-bp annotation-free regions are likely false. Only two possible false positive calls (0.04%) by NGS across all 100 samples (53-bp positions per sample x 100 samples = 5,300 bases) were detected. We conclude that, at least for the first coding exon of the *KRAS* gene, NGS is sensitive to identify true SNV while not generating a substantial number of false positives. Since both true positives and true negatives of somatic SNV in our tumor specimens are unknown, defining the actual sensitivity and specificity is left as an open question [40,41].

To further extend our findings about the sensitivity of NGS to detect mutations detected by Sanger sequencing, we analyzed a second gene *TP53* that has reported SNV distributed much more broadly than *KRAS*. Within the 544-bp region that harbors *TP53* (*RefGene ID*: *NM_000546*) exons 5, 6, 7, and 8, 317-bp positions have been annotated as variants by OncoMap plus COSMIC system or dbSNP, whereas 227-bp positions have no such annotation. Similar to the case of *KRAS*, we detected all 30 SNV that were independently detected by Sanger sequencing and only 20 possible false positive calls in the 227-bp annotation-free positions across all 100 samples (227 variant-free bp positions per sample x 100 samples = 22,700 bases). Interestingly, one of these 20 positive calls (Sample ID: 4 at *chr17*:*7578538*, *TP53*:*NM_000546*:*exon5*:*c*.*A392T*) was confirmed by Sanger sequencing to be a true positive. Although this position is not annotated for any SNV in lung by the COSMIC database (v66 or v72, the latest version during paper preparation) nor dbSNP, it is annotated in other cancer types, such as breast, central nervous system, liver, pancreas, and the upper aerodigestive and urinary tracts (COSMIC, v72). The other 19 calls have no Sanger sequencing data and are possible false positives (0.08%).

### Identification of Other Coexistent SNV and Indel by NGS

**Fig 4:**
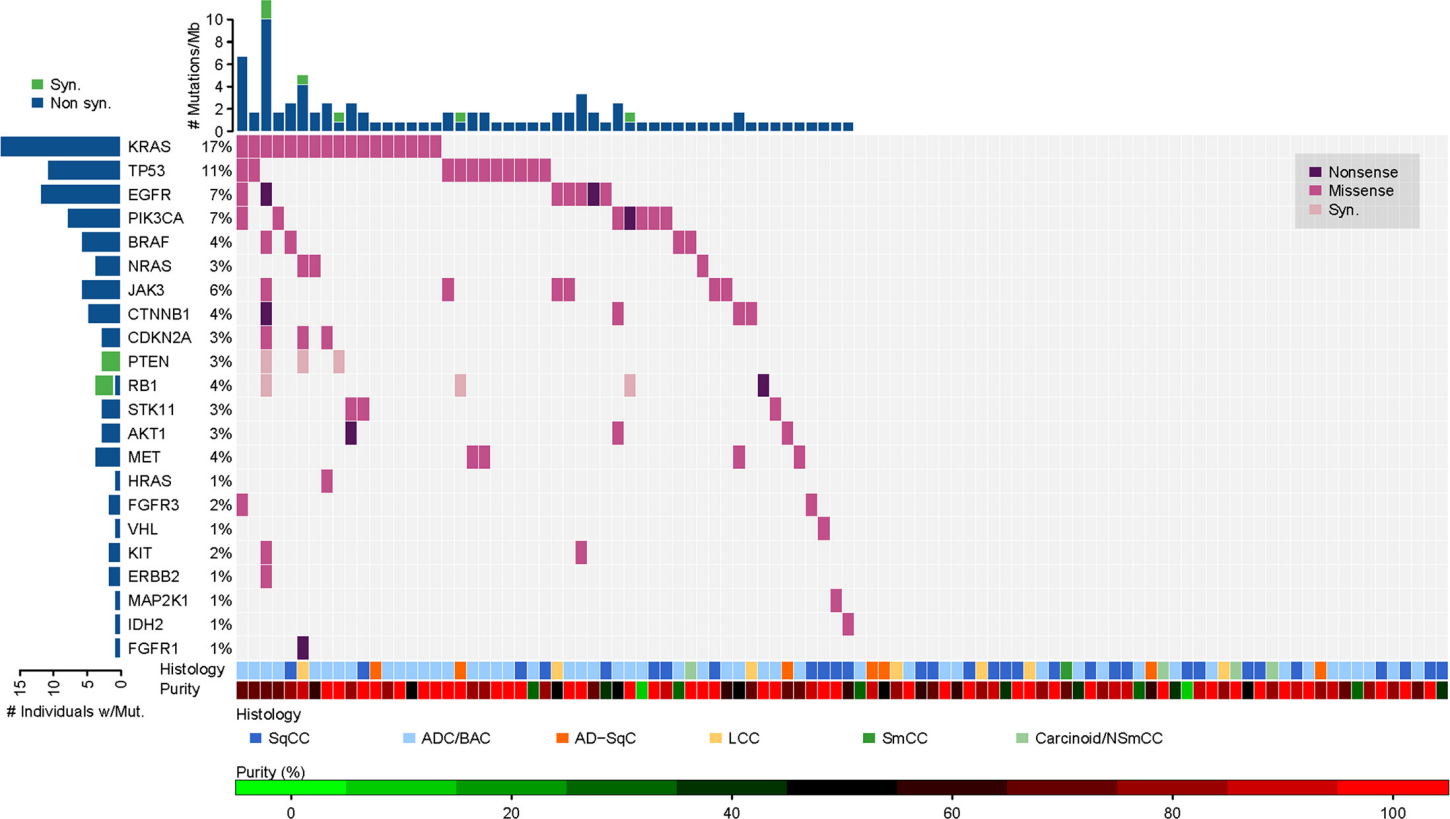
SNV Calling in Lung Cancer Specimens Using the UNCseq Assay for SNV Listed in the OncoMap System (‘Conservative’ SNV). Percentage, actual number of significantly mutated genes, and particular SNV types, nonsynonymous (nonsense, missense) and synonymous, are shown for each tumor sample in relation to its tumor histology and tumor purity. Abbreviations: SqCC: Squamous Cell Carcinoma; SmCC: Small Cell Carcinoma; ADC/BAC: Adenocarcinoma or Bronchio-alveolar Carcinoma; LCC: Large Cell Carcinoma; AD-SqC: Adenosquamous Carcinoma or Combined/Mixed; Carcinoid/NSmCC: Carcinoid-Atypical, Carcinoid-Typical, or Non-small cell carcinoma.

The power of NGS is the ability to detect genetic aberrations in large sets of genes, or even the entire genome, in a cost-effective fashion. We examined SNV in the 168 targeted genes that were consistently captured in both versions of our assay. Due to lack of matched germline information from our cases, we used prior knowledge from existing SNV annotation databases to refine mutations called using pooled normal. As shown in Fig 4 and using the deepSNV mutation calling method in OncoMap system, we detected 22 genes that were found to be significantly mutated (FDR ≤ 0.05) in our dataset (overall 101 mutations, approximately one SNV per sample). 49 tumor samples did not have any mutations in any of the 22 genes. 9 genes were the most frequently mutated (*KRAS*, *TP53*, *EGFR*, *PIK3CA*, *BRAF*, *NRAS*, *JAK3*, *CTNNB1*, *CDKN2A*) whereas 19 genes from the OncoMap database showed no SNV (*ABL1*, *AKT2*, *APC*, *CDK4*, *CSF1R*, *FGFR2*, *FLT3*, *GNA11*, *GNAQ*, *GNAS*, *IDH1*, *JAK2*, *MLH1*, *MYC*, *NPM1*, *PDGFRA*, *PIK3R1*, *RET*, *SRC*). Similar refined gene lists were obtained with VarScan2 (version 2.3.6) [42], and MuTect (version 1.1.4) [35]. The concordance rate for mutations within the OncoMap plus COSMIC system among deepSNV, MuTect, and VarScan2 was 100% (data not shown). Given the fact that we have performed Sanger sequencing in selected exons in a limited number of genes (*BRAF*, *CDKN2A*, *EGFR*, *STK11*, *TP53*) we have been unable to confirm indels identified by NGS.

Using the pooled normal control to identify SNV in our tumor samples against the ‘less-conservative’ list, we identified 42 significantly mutated genes (FDR ≤ 0.05) out of the total 168 targeted genes (S1 Fig). Remarkable was the hypermutated tumor (39 mutations) from patient 1 who was a smoker and had mutation in two DNA repair genes, as previously described [43]. Interestingly, a higher fraction of non-synonymous SNV was identified in OncoMap system (95/101 = 94.1% non-synonymous out of all polymorphisms) compared to OncoMap plus COSMIC system (292/364 = 80.2% non-synonymous out of all polymorphisms), indicating that the OncoMap system focuses more on functional-important variants. As shown in Table 2, the majority of genes that were previously shown to have non-synonymous SNV in previously published large datasets for lung squamous and lung adenocarcinoma [44, 45] were also present in our cohort using the OncoMap and COSMIC systems. In addition, new non-synonymous SNV were identified that were not previously described for both systems. However, we observed significantly lower frequency of SNV calling between OncoMap and the published datasets by individually comparing SNV call by OncoMap with those by each of the two published datasets. We obtained *p*-value < 0.05 in both comparison by paired permutation tests with exact *p*-value using the R function perm.test from the open source R package exactRankTests (http://cran.r-project.org/package=exactRankTests), under the null hypothesis that SNV calls are identically distributed for each SMG between OncoMap and the published dataset.

**Table 2:** SNV Calling (07–0120 Cohort) Using Two Different SNV Databases (OncoMap ± COSMIC) for the 2 most frequent histologic lung cancer types, adenocarcinoma and squamous carcinoma.

**Adenocarcinoma N = 50**	**OncoMap (%)**	**OncoMap plus COSMIC (%)**	**Imielinski Et Al (%)**
*TP53*	16	42	52
*KRAS*	26	28	27
*EGFR*	10	22	19
*STK11*	4	18	15
*KEAP1*	N/A	N/A	12
*ATM*	N/A	10	11
*NF1*	N/A	12	14
*SMARCA4*	N/A	8	13
*ARID1A*	N/A	0	9
*BRAF*	4	4	8
*RBM10*	N/A	N/A	7
*SETD2*	N/A	0	5
*PIK3CA*	10	18	5
*CBL*	N/A	0	4
*FBXW7*	N/A	16	4
*PPP2R1A*	N/A	0	4
*RB1*	6	12	3
*SMAD4*	N/A	8	4
*CTNNB1*	6	10	3
*U2AF1*	N/A	0	3
*KIAA0427*	N/A	N/A	2
*PTEN*	4	8	3
*BRD3*	N/A	N/A	2
*FGFR3*	2	0	2
*GOPC*	N/A	0	1
**Squamous Cell N = 31**	**OncoMap (%)**	**OncoMap plus COSMIC (%)**	**TCGA (%)**
*TP53*	6	55	81
*CDKN2A*	0	3	15
*PTEN*	0	0	8
*PIK3CA*	6	10	16
*KEAP1*	N/A	N/A	12
*MLL2*	N/A	0	20
*HLA-A*	N/A	N/A	3
*NFE2L2*	N/A	10	15
*NOTCH1*	N/A	0	8
*RB1*	0	10	7

Only significantly mutated genes that were identified in previously published databases [44, 45] are shown. Percentages indicate frequencies of mutated genes.

Indel analysis showed 23 deletions (14 nonframeshift and 9 frameshift) and no insertions. As shown in S1 Fig, we observed recurrent nonframeshift deletions in the *ARID4B* gene (*chr1*:*235377279–235377281*, p.548_549del), which have not been previously reported. These indel do not overlap with OncoMap and only one overlaps with OncoMap plus COSMIC system (*TP53*, *chr17*:*7579547–7579548*). No indel were noted in *ERBB2* and *EGFR*.

Finally, the 07–0120 cohort included less frequent histologic lung cancer subtypes, such as large-cell, adenosquamous, and carcinoid. Despite the small numbers in these rare lung cancer subtypes, SNV calling using the OncoMap database revealed previously undescribed SNV in *FGFR1*, *CDKN2A*, *RB1*, *JAK3*, and *CTNNB1* for the large-cell type, a *BRAF* mutation for carcinoid, and an *AKT1* mutation for adenosquamous carcinoma.

### Validation of SNV by RNA-Seq in the 07–0120 Patient Samples

We recently reported that RNA-seq data supports the detection of SNV called by DNA samples [31]. Therefore, we sequenced mRNA for 23 out of the total 100 samples to validate SNV previously called by DNA-seq. These 23 DNA samples exhibited a total of 79 significant SNV in OncoMap and the COSMIC database. 67 out of the 79 SNV (85%) were covered by at least one RNA-seq read. We found that the power to detect these SNV is in line with the expected mutant allele count (EMAC, or *E*) by RNA-seq, which is determined by the depth of RNA-seq and the MAF by DNA-seq (S1 Text). The confirmation rate is 86.3%, 92.9% and 100% for *E* equals to 1, 2 and 3, respectively (S2 Fig). For example, RNA-seq data was available for 8 out of 16 *KRAS*-hotspot-mutant samples, which were supported by either DNA or Sanger sequencing (one SNV per sample). Of these, 7 were confirmed by RNA-seq. The confirmation rate dropped to 6 out of 45 (13%) for SNV with *E* < 1. Of these 39 unconfirmed SNV (*E* < 1), 34 (87.2%) had DNA-seq MAF < 0.05, whereas 24 (61.5%) exhibited RNA-seq coverage depth < 5 (S2 Fig). Of the 19 samples that were reported to contain indels, only 6 had RNA-seq data, but with ≤ 6 reads crossing in the indel positions and ± 5-bp flanking regions. Therefore, we were unable to confirm indels previously identified by DNA-seq.

### NGS SNV Calling by Matched Germline DNA Versus Pooled Normal DNA and CLIA-certified Laboratory Confirmation in the 11–1115 Patient Samples

In clinical practice, a handful of NGS SNV calling algorithms have been frequently used in a CLIA environment and without matched germline DNA [3, 6]. However, to our knowledge, there has not been a systematic comparison between pooled normal, tumor-only variant calling, and the ‘gold-standard’, matched-normal. In line with this, we have investigated the consistency in SNV calling by the pooled normal, tumor-only variant calling versus matched-normal in clinical tumor tissue samples that were prospectively collected along with matched normal from patients with NSCLC (11–1115 cohort). To date, we have sequenced 24 tumor tissues (13 FFPE, 11 SF) from patients with NSCLC for diagnostic purposes and treatment decisions, and have performed independent CLIA-certified laboratory confirmation on the SNV identified by matched normal comparisons. We were able to retrieve 69.3% (median; approx. 95CI, 58.6–72.3%) and 69.6% (median; approx. 95CI, 59.7–72.0%) on-target bases with our targeted panel capture strategy of ClinSeq version 7 corresponding to tumor and matched-normal specimens, respectively. This was an improvement from ClinSeq version 5; under this version primer design was improved utilizing techniques suggested by the vendor (Agilent), including duplicate coverage of difficult to sequence regions (e.g. tiling). The median of the per-sample median RPKM was 350 (approx. 95CI, 344–353) and 347 (95CI, 344–354) for tumor and matched-normal specimens, respectively.

S3 Table shows NGS variant calling and CLIA confirmation of mutations either identified in the COSMIC and OncoMap databases or considered clinically important for genes with MAF ≥ 0.05. 17 out of the total 24 tumor samples exhibited a total of 35 nonsynonymous SNV/stopgain/indel when compared against matched-normal. Of the 35 mutations, 16 were selected and independently confirmed by a CLIA-certified laboratory. All SNV identified by either calls against matched-normal or pooled normal were also in agreement with normal-free genotyping analysis (SAMtools/BCFtools). More SNV were called against pooled normal compared with matched normal (46 versus 35). Of the 11 discrepancies, 7 nonsynonymous SNV were benign [PolyPhen-2 (PPH2) score ≤ 0.004] [46], 2 nonsynonymous SNV were possibly damaging (PPH2 <0.8), and 1 stopgain mutation had low MAF (0.06). The only probably damaging (PPH2 = 0.999) mutation that was identified again the pooled normal as opposed to the matched-normal was confirmed to be a germline mutation under the NCGENES project [47].

### Copy Number Variations

**Fig 5:**
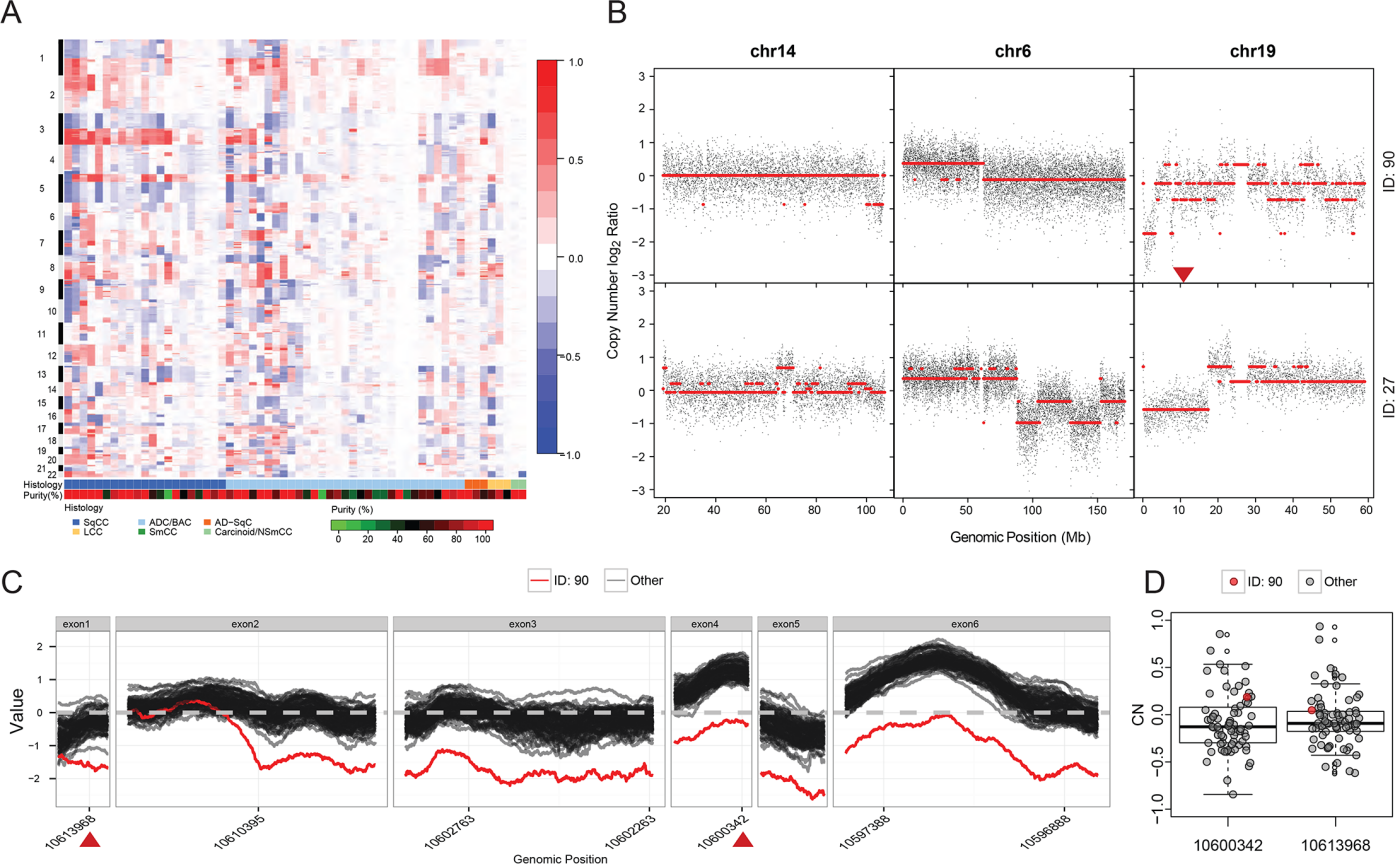
DNA Copy Number Analysis Using Affymetrix Human SNP Array 6.0 Microarray (Panel A) and UNCseq (Panel B, C) or Both (Panel D) of Lung Cancer Samples. **(A)** Copy number gains (red) and losses (blue) are plotted along the normal genome per each chromosome for each of the 60 completed tumor samples in relation to tumor histology and tumor purity. (SqCC: Squamous Cell Carcinoma; SmCC: Small Cell Carcinoma; ADC/BAC: Adenocarcinoma or Bronchio-alveolar Carcinoma; LCC: Large Cell Carcinoma; AD-SqC: Adenosquamous Carcinoma or Combined/Mixed; Carcinoid/NSmCC: Carcinoid-Atypical, Carcinoid-Typical, or Non-small cell carcinoma) **(B)** Examples of chromosome-level CNV in various chromosomes (6, 14, and 19) using UNCseq in two tumor samples (27 and 90). Black dots represent the per nucleotide relative copy number ratios (CNRs) in log_2_. Segmentation-derived regions of equal copy number are indicated in red lines. A red triangle at 10.6 Kbp position of chromosome 19 in the sample (ID: 90) indicates the zoomed regions in panel C. **(C)** Example of small (gene-level) structural variations across exons (from 5’ to 3’) of the *KEAP1* gene (RefGene ID: *NM_203500*) for all (black) but one (red) tumor samples. Markers from the Genome-Wide Human SNP Array 6.0 corresponding to the chromosome area where *KEAP1* gene is located are highlighted in red triangles. **(D)** Boxplot analysis illustrating the SNP array signals at these two markers in C. Signals of tumor sample 90 are in red.

Technologies estimating copy number variations (CNV) prior to NGS could reliably assess large (several Kbps to Mbps) gains or losses of DNA regions [48]. Due to the nature of NGS, which detects genetic events with 1-bp resolution, we used flexible window size to detect not only chromosome arm-level events commonly detected by other technologies, but also CNV at the single gene level [49]. Fig 5, panel A, shows SNP array analysis for CNV, which was performed on a subset (n = 60) of the 07–0120 tumor samples that were also subjected to TPS. In this analysis we were able to detect previously described chromosome arm-level events, such as 1p loss and 1q gain in lung squamous and adenocarcinomas, as well as 3p loss and 3q gain in lung squamous only [44, 45]. We then compared CNV detection tools that are either NGS-based, such as VarScan and *NGScopy*, or non-NGS-based, such as SNP array. As shown in S3 Fig, using SNP array and *NGScopy* we clearly saw 6p amplification in tumor ID 90 (07–0120 cohort). However, this chromosomal arm aberration was not clear when VarScan2 was applied. Fig 5, panel B, shows examples of CNV in several chromosomes for a given tumor sample using both *NGScopy* and SNP array. Similar to SNP array, *NGScopy* can equally detect chromosome arm-level CNV. Fig 5, panel B, shows different CNV patterns (e.g. copy neutrality, gain, loss, or chromosome fragmentation) that can be detected with *NGScopy* in different samples.

To show that *NGScopy* can detect CNV at the gene level, we analyzed the 17,259-bp chromosome region (*chr19*:*10*,*596*,*796–10*,*614*,*054*) that contains the 6 exons of the Kelch-Like ECH-Associated Protein 1 (*KEAP1*) gene, which can undergo intragenic CNV [50] (S4 Fig). Fig 5, panel C, shows relative copy number (in log_2_) of all nucleotides within each of the 6 exons of the *KEAP1* gene across 64 tumor samples with available NGS data using ClinSeq4. Almost all tumor samples do not bear intragenic CNV across all 6 exons of the *KEAP1* gene. However, a single tumor specimen (case 90) shows loss of most exonic regions of the *KEAP1* gene, except the 3’ area of exon 2 (S4 Fig). This gene-level copy number loss would have been extremely difficult to be detected by the SNP array technology, as only two probes cover the corresponding region. In fact, both SNP probes at locations 10,600,342 and 10,613,968, which are within exons 1 and 4 of the *KEAP1* gene, respectively, show slight gain, if any, in both locations (Fig 5, panels C, and D). Our results indicate that NGS is more sensitive in detecting intragenic structural genetic variations than the SNP array.

## Discussion

In this report, we present the UNC-CH experience and performance of an NGS-based TPS assay, termed UNCseq, to analyze lung cancer samples. During the early developmental steps of this assay, we previously showed that we can identify genetic events in DNA obtained from cancer cell lines and SF tumors [24]. We now present methodological details, in particular the downstream bioinformatics pipeline, and extend our experience to a well clinicopathologically annotated, retrospectively collected lung cancer tumor specimen collection (07–0120 tumor cohort). We have validated important mutations in lung cancer, such as *KRAS* and *TP53*, using Sanger sequencing in a research (i.e. non CLIA-certified) laboratory environment. In addition, we have applied our assay along with an independent CLIA validation strategy of clinically relevant SNV in a separate, prospectively collected tumor tissue cohort that not only includes ‘real-world,’ FFPE, and tumor tissue samples, but for which there is available germline DNA (11–1115 tumor cohort). Finally, we apply our recently published RNAseq-based bioinformatics method, UNCeqR [31], to validate SNV identified by DNA-seq.

Similar to other reports, we have shown that NGS is more sensitive than Sanger sequencing in detecting somatic hot-spot SNV of the *KRAS* gene [51, 52]. In our dataset, the increased sensitivity of NGS over Sanger sequencing was mainly attributed to its ability to detect low-frequency tumor clones bearing SNV of interest, whereas stromal contamination > 50% or coverage depth < 100x did not play a significant role. Depth of coverage in DNA sequencing is one of the most important parameters affecting cost, computational time, and ultimately speed to report results back to the patient [53]. Recent reports have recommended that > 200x coverage is required to maintain high sensitivity in detecting low frequency base substitutions [3] and > 30x coverage was sufficient to detect mutations at allele fractions ≥ 0.2 with approximately 90% sensitivity [35]. In the case of detecting low-MAF *KRAS* hotspot SNV using UNCseq, this was indeed true; none of the 4 samples that exhibited approximately or less than 5% MAF (Fig 3C) had NGS coverage less than 100x. Nevertheless, in our study, we show that we were successful in achieving at least 50x coverage, the minimum requirement for reliable SNV calling [54], by loading 8 samples per lane for nearly all tumor samples, based on our TPS strategy and the performance of the HiSeq 2000 instrument.

Availability of germline DNA would increase analytical sensitivity and distinguish between inherited variant and somatic SNV [55], and lack of same-patient germline DNA significantly affects frequency of calling SNV. While the lack of germline DNA information is a weakness for the SNV calling in the 07–0120 tumor cohort, it is a common challenge faced in clinical cancer research and is currently widely used in every day clinical practice [3]. Nevertheless, there is insufficient knowledge about direct comparison between the gold standard (i.e. germline DNA control) with other mutation calling strategies, such SNV calling against pooled normal DNA or normal-free genotyping analysis. At least for SNV previously reported in known mutation databases, such as OncoMap and COSMIC, all mutations detected in the 24 tumor specimens using the germline DNA comparison algorithm were also detected in the exact same samples that were compared against pooled normal DNA. Furthermore, all mutations detected using the pooled normal DNA strategy were consistent with normal-free genotyping analysis. However, non-germline DNA-based mutation calling strategies were associated with over-reporting of SNV. From this direct comparison between these two SNV calling strategies (i.e. germline DNA versus pooled normal DNA), the ‘price’ paid for the lack of germline DNA information does not appear to be detrimental; two of the over-reported SNV using the pooled normal DNA strategy did not have significant impact in protein function, as assessed by the PolyPhen-2 algorithm, with all its limitations [56]. However, the third over-reported SNV in the 11–1115 cohort was clinically important because it was a germline mutation, which could not be distinguished from somatic mutations by the lack of matched normal samples.

Developing methodologies for SNV calling in tumor tissues lacking same-patient germline DNA using prior knowledge from mutation databases for diagnostic purposes, but not for pure discovery research, are important. Our SNV calling algorithm that was applied to the 07–0120 tumor cohort reveals that the more stringent the SNV database is, the lower the frequency of SNV calls. In particular, using the combined sequence information from both OncoMap and COSMIC databases for the 07–0120 tumor cohort, the frequency of SNV calls using our algorithm was not significantly different compared to large, published lung cancer datasets with available germline DNA [44, 45]. We also found that the significantly higher frequency of SNV calling using less stringent databases led to a significantly higher identification of clinically unimportant SNV (e.g., synonymous) or more non-synonymous SNV of unknown clinical significance. These findings from the 07–0120 tumor cohort, as well as the over-reporting of SNV that do not have significant impact in protein function using the pooled normal DNA as compared with the matched normal DNA in the 11–1115 tumor cohort, suggest that lack of same-patient germline DNA does not significantly affect frequency of calling of detrimental SNV.

Among other potential applications, UNCeqR allows for lack of germline DNA and provides the basis for additional validation of SNV called by DNA-seq [31]. Although tumor purity was not a particular challenge in our dataset (12% of tumor samples exhibited ≤ 40% tumor content), RNA-seq was unable to validate all mutations detected from DNA-seq due to low RNA depth and/or low DNA MAF. It is quite possible that higher confirmation rates might have been achieved if we had greater depth of RNA-seq. Also, due to the current study’s exploratory nature of RNA-seq as an independent SNV validation tool, we did not expand our investigation into using RNA-seq as an independent SNV detection algorithm for our samples. Nevertheless, given our prior analysis by methods that integrate same-tumor RNA and DNA-seq data, such as UNCeqR, we envision that identifying clinically important, ‘driver’ SNV in clinical specimens by combining RNA and DNA sequencing might enhance sensitivity and specificity and possibly lower overall cost.

While the clinical significance of large copy number variations in cancer is well established [57], the role of gene-level or even smaller scale (i.e. <1 Kbp) alterations in tumor development, progression, and treatment is largely unknown. Given the 1-bp resolution of NGS, development of bioinformatics methods to detect CNV, in particular structural DNA changes < 1Kbp, is appealing. Furthermore, data generated by TPS are particularly challenging to extrapolate for genome-wide CNV detection given the *de facto* uneven coverage across the genome. It is therefore not surprising that applications of TPS to the analysis of CNV are underdeveloped [58, 59]. In line with this unmet need, we have developed a CNV detection method that depends on the coverage depth in a tumor sample in comparison with pooled germline DNA, termed *NGScopy*. This method takes into account both on-target and off-target sequence reads, which are expected to be randomly distributed across the gene panel and the entire genome, respectively. Therefore, while these off-target reads do not contribute to SNV calling, they are valuable to compute genome-wide copy number events. Finally, this method relies on flexible window size as well as the distribution of the coverage depth in normal samples. Overall, we show that *NGScopy* detects large-scale CNV, similar to those identified by the genome-wide Affymetrix Human SNP Array 6.0, and performs better for TPS data compared to popular CNV NGS-based algorithm, such as VarScan. However, NGS, by having greatly improved resolution over SNP array, was superior in terms of detecting small-scale intragenic CNV. We propose that CNV detection tools using NGS data, such as *NGScopy*, could supplement CNV detection by SNP arrays, especially for the largely unexplored evaluation of small-scale sequencing (e.g., TPS) in cancer.

Despite the strength of our study to compare our NGS-based analysis with gold-standard approaches, such as Sanger sequencing and SNP arrays, there are several limitations. First, and in particular for the 07–0120 tumor cohort, we used the less expensive and more time-efficient single-end sequencing, as opposed to the more established paired-end sequencing. Single-end reads may pose computational challenges in the differential diagnosis and estimation of the percentage of PCR when compared to true biological duplicate reads [60]. Therefore, removal of duplicates may on one hand limit overestimation of true coverage depth, but may on the other hand eliminate other true alignments during further downstream analysis. In fact, for the 11–1115 tumor cohort, we have currently incorporated paired-end sequencing in routine sequencing as part of the UNCseq project. Second, like any NGS-based assay that is currently implemented, refinement and optimization of UNCseq assay is a moving target in several areas ranging from the number and type of genes to be analyzed, the targeted exome capture, the sequencing strategy (single-end versus paired end), and the downstream bioinformatics pipeline (S2 Table). In our study this is more apparent in the analysis of the 07–0120 tumor cohort for which two different gene lists were used (ClinSeq version 4 and 5). As the gene list is further updated, future ClinSeq versions are planned to include additional probes to also capture particular recurrent structural DNA alterations that we could not previously identify, such as genome rearrangements that in fact are frequently observed in lung cancer [61, 62].

In summary, we present the performance of our NGS-based laboratory assay, termed UNCseq, to detect genetic aberrations (SNV, indel, and CNV) in a large number of lung cancer specimens that were collected in our institution. We show that when compared to traditional methods, such as Sanger sequencing and the genome-wide Affymetrix Human SNP Array 6.0, TPS is even more sensitive in mutation calling at a 50-100x coverage, and may detect intragenic CNV that are not identified by SNP arrays. We further report that the majority of clinically relevant genes that were found in our study to be mutated were also observed in large previously published datasets [40, 41]. We provide direct comparison between germline and non-germline DNA-based mutation calling strategies using the UNCseq assay, as well as independent CLIA validation of several mutations in a separate tumor tissue cohort (11–1115). We believe that our UNCseq methodology is standardized, robust, and can provide valuable genetic information about clinically actionable mutations in a cost-effective and time-efficient manner, if paired with validation in a CLIA-certified laboratory.

## Supporting Information

S1 Fig SNV and indel calling in lung cancer specimens using the UNCseq assay. **(A)** SNV listed in the OncoMap annotation (version 4) plus the COSMIC database (“liberal” SNV). Percentage, actual number of significantly mutated genes, and particular SNV types [nonsynonymous (nonsense, missense) and synonymous] are shown for each tumor sample in relation to its tumor histology and tumor purity and colored differently for each of the two SNV databases (OncoMap, COSMIC). **(B)** Indel calling was performed without refining using OncoMap and COSMIC database.
(PDF)

S2 Fig Validation of SNV that were identified by DNA-seq and were covered by at least one RNA-seq read. The coverage depth by RNA-seq and the MAF by DNA-seq are shown, on logarithmic-scale axes, for each SNV of OncoMap and COSMIC. Circles and triangles indicate confirmed and unconfirmed SNV, respectively. *KRAS*, *TP53*, and *EGFR* which were found to be most frequently mutated in our sample set are highlighted with orange, purple, and blue, respectively. Dashed lines indicate expected mutant allele count (*E*) equaling to 1, 2 and 3.
(PDF)

S3 Fig Comparison of copy number variation calling using VarScan (A), *NGScopy* (B), and SNParray (C) on chromosome 6 for patient ID 90 (07–0120 cohort). Gray dots indicate the log_2_ copy number ratio of the tumor against the pooled normal per each window detected by each computational program. *NGScopy* and SNParray show clear 6p amplification compared with VarScan. The y-axis was truncated to [-3, 3] as we zoom in on a majority (99.42%, 100%, 99.96%, respectively) of the data for comparison.
(PDF)

S4 Fig Targeted panel sequencing of the KEAP1 gene for patient ID 90 (07–0120 tissue cohort). Panels show an adapted Omicsoft's genome browser view (http://www.omicsoft.com/genome-browser/). The chromosome position and the structure of the *KEAP1* isoforms of NCBI RefGene is shown in **(A)**. The depth of coverage using TPS for patient ID 90 (bottom) against the pooled normal (top) is shown in **(B)**. Read counts were normalized to Reads Per Ten Million (RPTM) reads per library. Red corresponds to covered exon regions and purple corresponds to covered intron/intergenic regions.
(PDF)

S1 Table List of genes that were included in ClinSeq versions 4, 5 and 7 of the UNCseq project. (DOCX)

S2 Table Key differences of the UNCseq sequencing, bioinformatics pipeline, and validation of tissue samples between the 07–0120 and 11–1115 cohort. *Abbreviations*: SF, snap-frozen; FFPE, formalin-fixed paraffin-embedded; bp, base pair; PCR, polymerase chain reaction; CLIA, clinical laboratory improvement amendments.
(DOCX)

S3 Table List of mutations identified in the 24 tumor tissue samples collected as part of the 11–1115 tissue cohort using three methods to call mutations (matched normal, pooled normal, and normal free variant calling). Abbreviations: SF, snap-frozen; FFPE, formalin-fixed paraffin-embedded; SNV, single-nucleotide variations; CLIA, clinical laboratory improvement amendments; SOC, standard of care assays used by CLIA-certified Molecular Pathology laboratories to report mutations for clinical use; PPH2, PolyPhen 2; MAF, mutant allele frequency; Nonsyn., Nonsynonymous; Nonframe., Nonframeshift; Frame., Frameshift; ins., insertion; del.,deletion; dam., damage.
(DOCX)

S1 Text Definitions for depth of coverage (or read depth), breath of coverage, on-target rate, and expected mutant allele count. (DOCX)
