# Supplementary figures and images for: Combined Targeted DNA Sequencing in Non-Small Cell Lung Cancer (NSCLC) Using UNCseq and NGScopy, and RNA Sequencing Using UNCqeR for the Detection of Genetic Aberrations in NSCLC
Source: PLoS One. 2015 Jun 15;10(6):e0129280. doi: 10.1371/journal.pone.0129280 (PMC4468211; doi:10.1371/journal.pone.0129280)

A

SNV

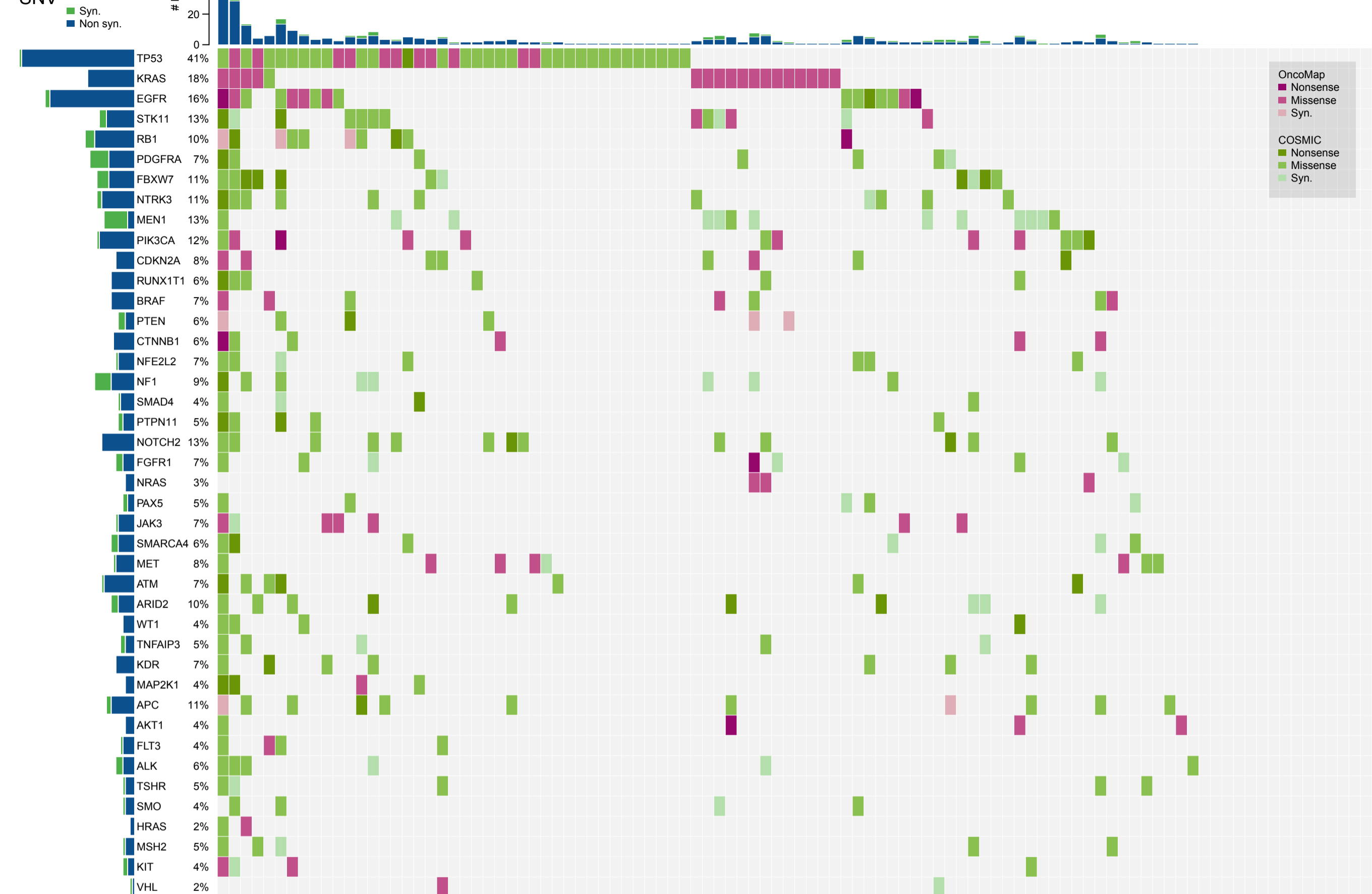

B

Indel

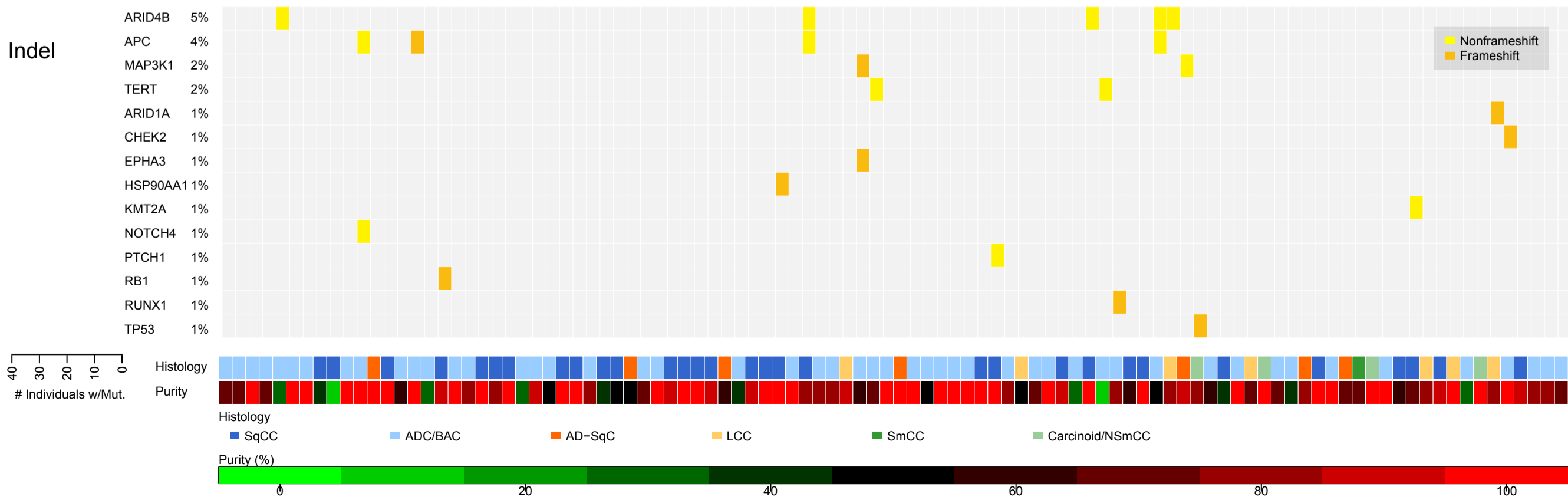

Supplement: S1 Fig — (A) SNV listed in the OncoMap annotation (version 4) plus the COSMIC database (“liberal” SNV). Percentage, actual number of significantly mutated genes, and particular SNV types [nonsynonymous (nonsense, missense) and synonymous] are shown for each tumor sample in relation to its tumor histology and tumor purity and colored differently for each of the two SNV databases (OncoMap, COSMIC). (B) Indel calling was performed without refining using OncoMap and COSMIC database. (PDF) [file pone.0129280.s001.pdf]

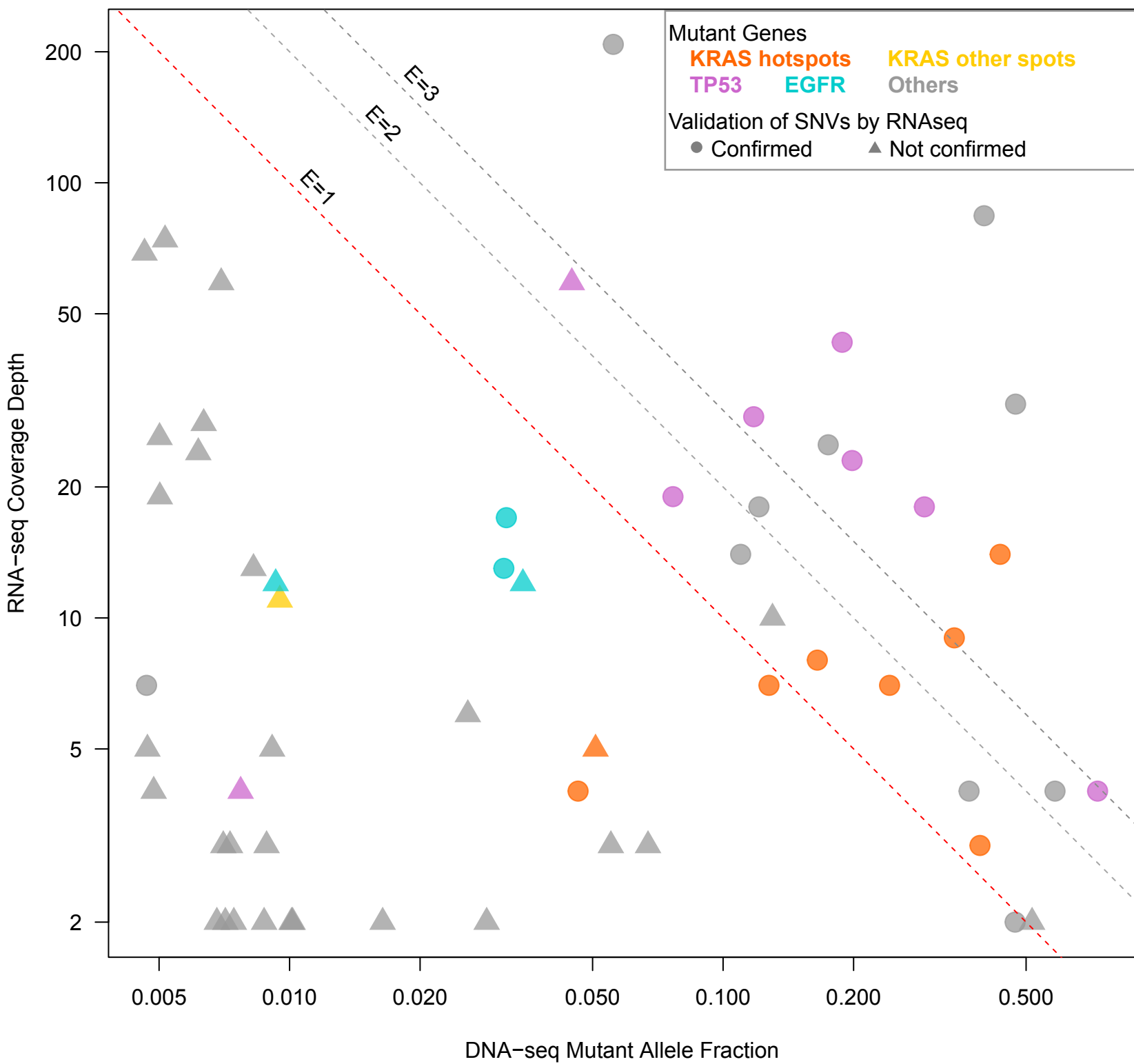

Supplement: S2 Fig — The coverage depth by RNA-seq and the MAF by DNA-seq are shown, on logarithmic-scale axes, for each SNV of OncoMap and COSMIC. Circles and triangles indicate confirmed and unconfirmed SNV, respectively. KRAS, TP53, and EGFR which were found to be most frequently mutated in our sample set are highlighted with orange, purple, and blue, respectively. Dashed lines indicate expected mutant allele count (E) equaling to 1, 2 and 3. (PDF) [file pone.0129280.s002.pdf]

Tumor (ID: 90) – VarScan

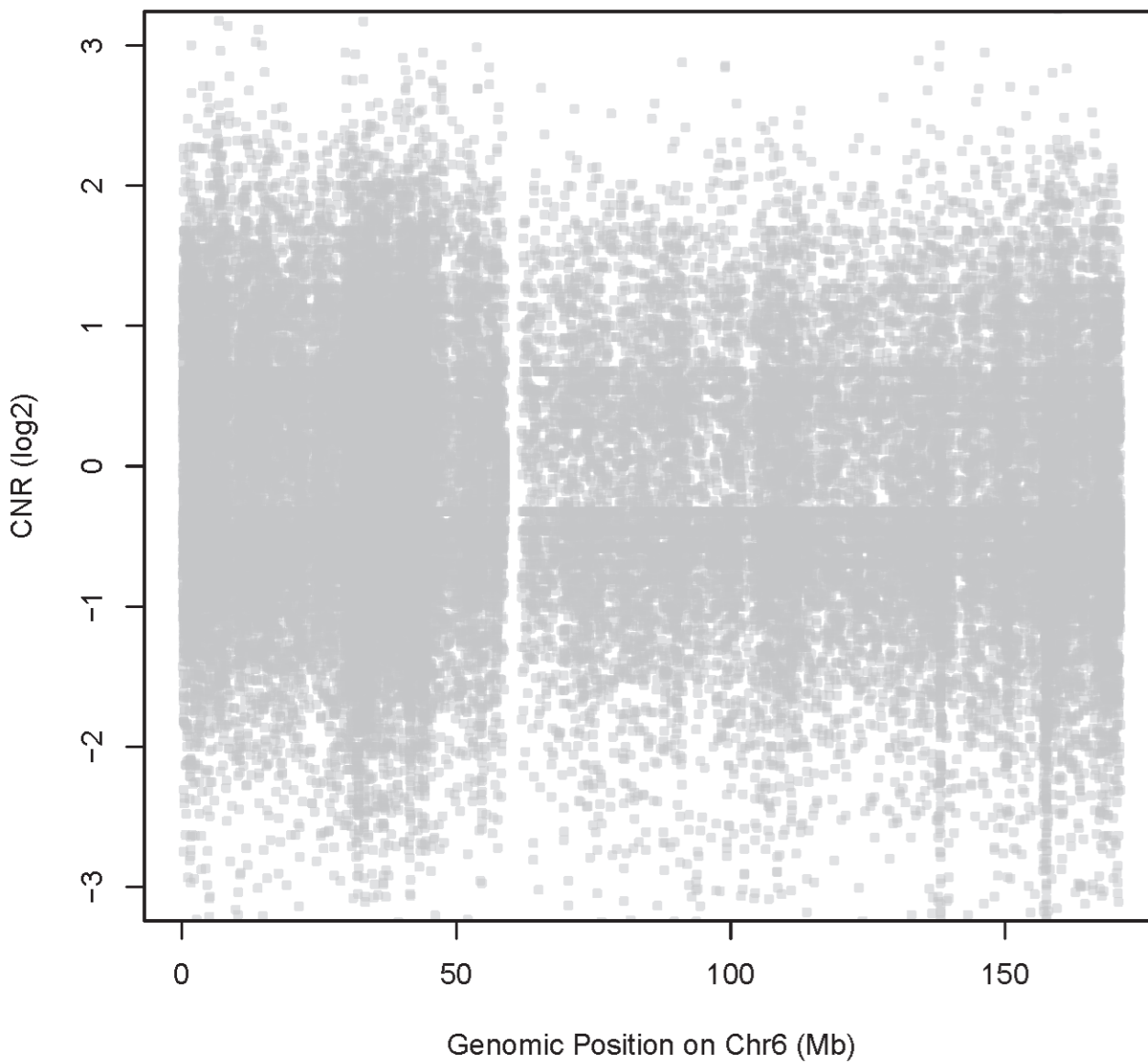

Tumor (ID: 90) – NGScopy

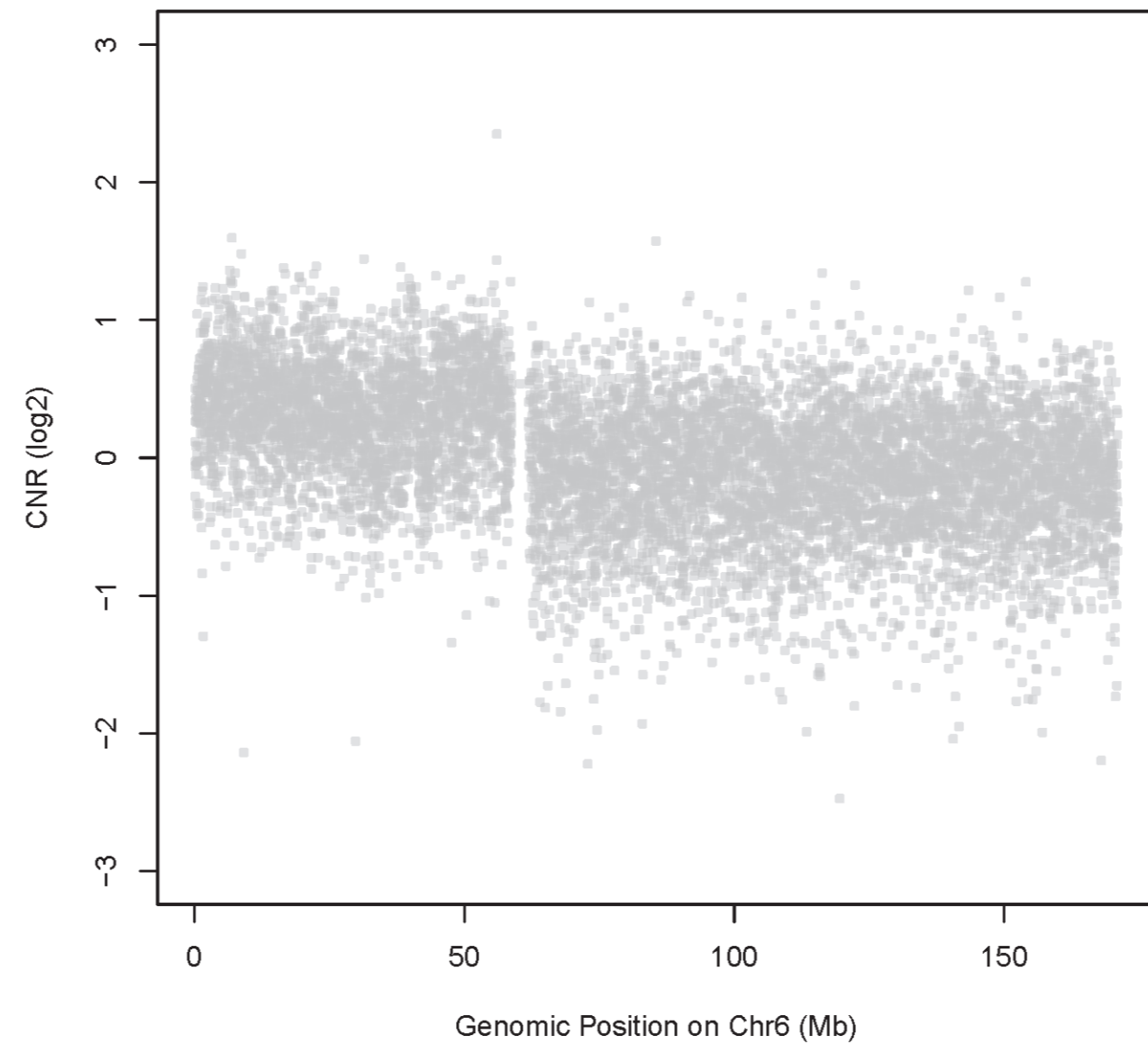

Tumor (ID: 90) – SNParray

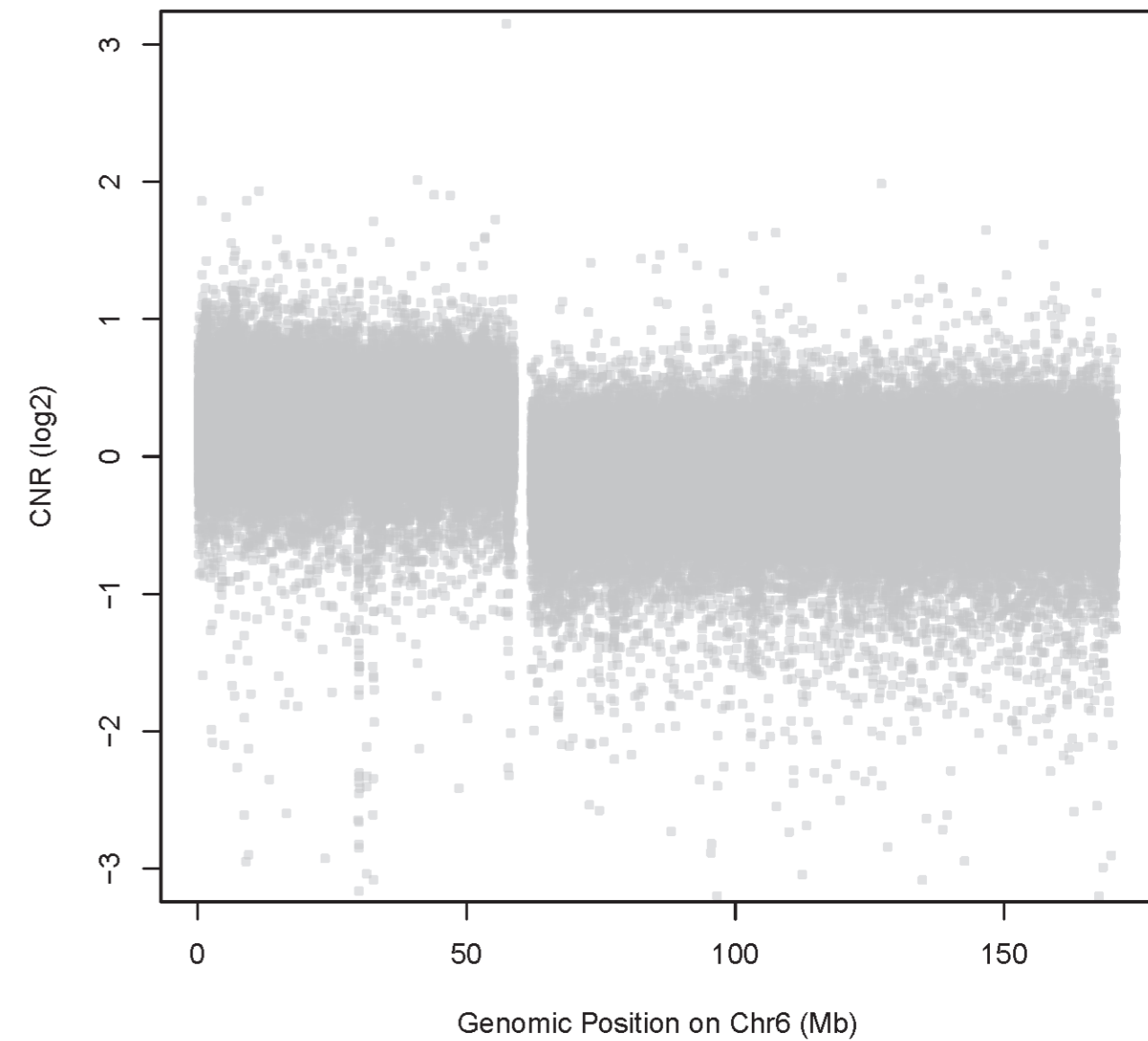

Supplement: S3 Fig — Gray dots indicate the log2 copy number ratio of the tumor against the pooled normal per each window detected by each computational program. NGScopy and SNParray show clear 6p amplification compared with VarScan. The y-axis was truncated to [-3, 3] as we zoom in on a majority (99.42%, 100%, 99.96%, respectively) of the data for comparison. (PDF) [file pone.0129280.s003.pdf]

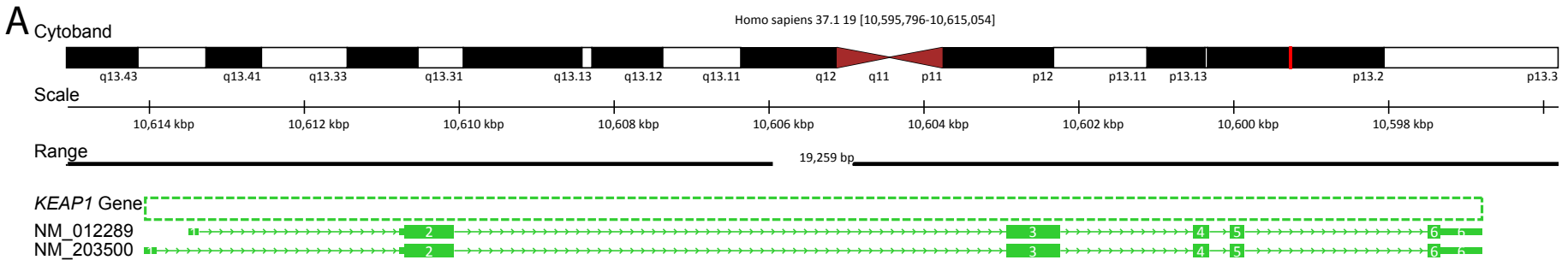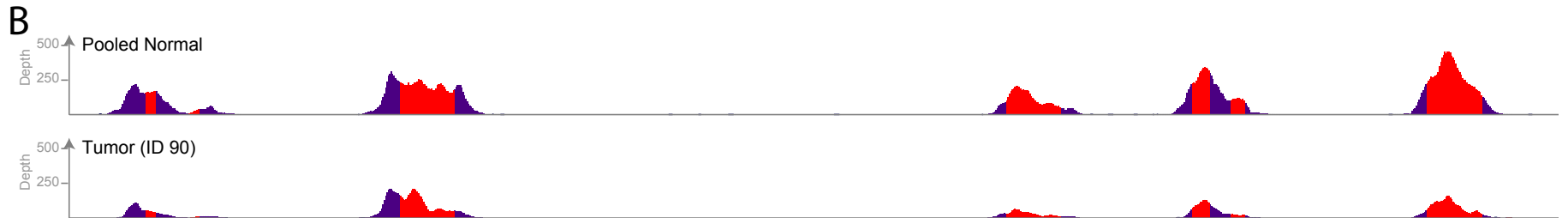

Supplement: S4 Fig — Panels show an adapted Omicsoft's genome browser view (http://www.omicsoft.com/genome-browser/). The chromosome position and the structure of the KEAP1 isoforms of NCBI RefGene is shown in (A). The depth of coverage using TPS for patient ID 90 (bottom) against the pooled normal (top) is shown in (B). Read counts were normalized to Reads Per Ten Million (RPTM) reads per library. Red corresponds to covered exon regions and purple corresponds to covered intron/intergenic regions. (PDF) [file pone.0129280.s004.pdf]
