# Supplementary material for: Combined Targeted DNA Sequencing in Non-Small Cell Lung Cancer (NSCLC) Using UNCseq and NGScopy, and RNA Sequencing Using UNCqeR for the Detection of Genetic Aberrations in NSCLC
Source: PLoS One. 2015 Jun 15;10(6):e0129280. doi: 10.1371/journal.pone.0129280 (PMC4468211; doi:10.1371/journal.pone.0129280)
Supplement: S1 Table — (DOCX) [file pone.0129280.s005.docx]

|  | Gene | ClinSeq Version 4 5 7 | | |
| --- | --- | --- | --- | --- |
| 1 | *ABCB1* |  |  | * |
| 2 | *ABL1* | * | * | * |
| 3 | *ABL2* | * | * | * |
| 4 | *ACRV1* | * |  |  |
| 5 | *ADRB1* |  |  | * |
| 6 | *ADRB2* |  |  | * |
| 7 | *AGPS* | * |  |  |
| 8 | *AKT1* | * | * | * |
| 9 | *AKT2* | * | * | * |
| 10 | *AKT3* | * | * | * |
| 11 | *ALK* | * | * | * |
| 12 | *ALOX5* |  |  | * |
| 13 | *ANKRD49* | * |  |  |
| 14 | *APC* | * | * | * |
| 15 | *APC2* |  |  | * |
| 16 | *AR* | * | * | * |
| 17 | *ARID1A* | * | * | * |
| 18 | *ARID1B* | * | * | * |
| 19 | *ARID2* | * | * | * |
| 20 | *ARID3A* | * | * | * |
| 21 | *ARID3B* | * | * | * |
| 22 | *ARID4A* | * | * | * |
| 23 | *ARID4B* | * | * | * |
| 24 | *ARID5A* | * | * | * |
| 25 | *ARID5B* | * | * | * |
| 26 | *ASXL1* |  |  | * |
| 27 | *ATM* | * | * | * |
| 28 | *ATR* |  | * | * |
| 29 | *AURKA* | * | * | * |
| 30 | *BCL2* | * | * | * |
| 31 | *BCR* |  |  | * |
| 32 | *BRAF* | * | * | * |
| 33 | *BRCA1* | * | * | * |
| 34 | *BRCA2* | * | * | * |
| 35 | *BRD4* |  |  | * |
| 36 | *CBL* |  |  | * |
| 37 | *CBLB* |  |  | * |
| 38 | *CCND1* | * | * | * |
| 39 | *CCNE1* | * | * | * |
| 40 | *CDC73* | * | * | * |
| 41 | *CDH1* | * | * | * |
| 42 | *CDH6* |  | * | * |
| 43 | *CDK4* | * | * | * |
| 44 | *CDK6* | * | * | * |
| 45 | *CDK8* | * | * | * |
| 46 | *CDKN1A* | * | * | * |
| 47 | *CDKN1B* | * | * | * |
| 48 | *CDKN2A* | * | * | * |
| 49 | *CDKN2B* |  | * | * |
| 50 | *CDKN2B-AS* | * |  |  |
| 51 | *CEBPA* | * | * | * |
| 52 | *CHD5* | * | * | * |
| 53 | *CHD6* | * |  |  |
| 54 | *CHEK1* | * | * | * |
| 55 | *CHEK2* | * | * | * |
| 56 | *COBRA1* |  |  | * |
| 57 | *COMT* |  |  | * |
| 58 | *CREBBP* | * | * | * |
| 59 | *CRKL* | * | * | * |
| 60 | *CSDE1* | * |  |  |
| 61 | *CSF1R* | * | * | * |
| 62 | *CSTF1* | * |  |  |
| 63 | *CTNNB1* | * | * | * |
| 64 | *CYP2A6* |  |  | * |
| 65 | *CYP2B6* |  |  | * |
| 66 | *CYP2C19* |  |  | * |
| 67 | *CYP2C8* |  |  | * |
| 68 | *CYP2C9* |  |  | * |
| 69 | *CYP2D6* |  |  | * |
| 70 | *CYP3A4* |  |  | * |
| 71 | *CYP3A5* |  |  | * |
| 72 | *CYP4F2* |  |  | * |
| 73 | *DERL3* | * |  |  |
| 74 | *DNMT3A* |  |  | * |
| 75 | *DPYD* |  |  | * |
| 76 | *DRD2* |  |  | * |
| 77 | *EGFR* | * | * | * |
| 78 | *EPHA10* | * | * | * |
| 79 | *EPHA3* | * | * | * |
| 80 | *EPHA5* | * | * | * |
| 81 | *EPHA6* | * | * | * |
| 82 | *EPHB6* | * | * | * |
| 83 | *EPM2AIP1* | * |  |  |
| 84 | *ERBB2* | * | * | * |
| 85 | *ERBB3* | * | * | * |
| 86 | *ERBB4* | * | * | * |
| 87 | *ERCC1* |  | * | * |
| 88 | *ERG* |  |  | * |
| 89 | *FAM123B* | * | * | * |
| 90 | *FBXO11* | * |  |  |
| 91 | *FBXW7* | * | * | * |
| 92 | *FGFR1* | * | * | * |
| 93 | *FGFR2* | * | * | * |
| 94 | *FGFR3* | * | * | * |
| 95 | *FGFR4* | * | * | * |
| 96 | *FHIT* | * | * | * |
| 97 | *FIP1L1* | * |  |  |
| 98 | *FKBP1B* | * |  |  |
| 99 | *FKBP9* | * | * | * |
| 100 | *FLJ16341* | * |  |  |
| 101 | *FLJ31306* | * |  |  |
| 102 | *FLJ37453* | * |  |  |
| 103 | *FLJ42627* | * |  |  |
| 104 | *FLT1* | * | * | * |
| 105 | *FLT3* | * | * | * |
| 106 | *FLT4* | * | * | * |
| 107 | *FOLR1* |  | * | * |
| 108 | *FZR1* | * |  |  |
| 109 | *G6PD* |  |  | * |
| 110 | *GATA3* |  |  | * |
| 111 | *GGPS1* | * |  |  |
| 112 | *GNA11* | * | * | * |
| 113 | *GNAQ* | * | * | * |
| 114 | *GNAS* | * | * | * |
| 115 | *GNAS-AS1* | * |  |  |
| 116 | *GNL3* | * |  |  |
| 117 | *GPSM3* | * |  |  |
| 118 | *GSTM1* |  |  | * |
| 119 | *GSTP1* |  |  | * |
| 120 | *GSTT1* |  |  | * |
| 121 | *GUCY1A2* | * | * | * |
| 122 | *H3F3A* |  |  | * |
| 123 | *H3F3B* |  |  | * |
| 124 | *HECW1* | * | * | * |
| 125 | *HLA-A* |  |  | * |
| 126 | *HLA-B* |  |  | * |
| 127 | *HMGXB3* | * |  |  |
| 128 | *HNF1A* | * | * | * |
| 129 | *HRAS* | * | * | * |
| 130 | *HSCB* | * |  |  |
| 131 | *HSP90AA1* | * | * | * |
| 132 | *IDH1* | * | * | * |
| 133 | *IDH2* | * | * | * |
| 134 | *IGF1R* | * | * | * |
| 135 | *IKBKE* | * | * | * |
| 136 | *IKZF1* | * | * | * |
| 137 | *IL28B* |  |  | * |
| 138 | *INSRR* | * |  |  |
| 139 | *ITPA* |  |  | * |
| 140 | *JAK1* |  | * | * |
| 141 | *JAK2* | * | * | * |
| 142 | *JAK3* | * | * | * |
| 143 | *JARID2* |  |  | * |
| 144 | *KCNH2* |  |  | * |
| 145 | *KCNJ11* |  |  | * |
| 146 | *KDM5A* |  |  | * |
| 147 | *KDM5B* |  |  | * |
| 148 | *KDM5C* |  | * | * |
| 149 | *KDM6A* | * | * | * |
| 150 | *KDR* | * | * | * |
| 151 | *KEAP1* | * | * | * |
| 152 | *KIT* | * | * | * |
| 153 | *KLLN* | * |  |  |
| 154 | *KRAS* | * | * | * |
| 155 | *LOC100130691* | * |  |  |
| 156 | *LOC100144602* | * |  |  |
| 157 | *LOC100506895* | * |  |  |
| 158 | *LOC100507346* | * |  |  |
| 159 | *LOC283738* | * |  |  |
| 160 | *LOC407835* | * |  |  |
| 161 | *LOC652276* | * |  |  |
| 162 | *LOC80054* | * |  |  |
| 163 | *LRRFIP2* | * |  |  |
| 164 | *MAP2K1* | * | * | * |
| 165 | *MAP2K2* | * | * | * |
| 166 | *MAP2K4* | * | * | * |
| 167 | *MAP3K1* | * | * | * |
| 168 | *MAP3K8* | * | * | * |
| 169 | *MCL1* | * | * | * |
| 170 | *MDM2* | * | * | * |
| 171 | *MDM4* | * | * | * |
| 172 | *MEN1* | * | * | * |
| 173 | *MERTK* |  |  | * |
| 174 | *MET* | * | * | * |
| 175 | *MIEN1* |  |  | * |
| 176 | *MIR1204* | * |  |  |
| 177 | *MIR4673* | * |  |  |
| 178 | *MIR4674* | * |  |  |
| 179 | *MIR4728* | * |  |  |
| 180 | *MITF* | * | * | * |
| 181 | *MKRN2* | * |  |  |
| 182 | *MLH1* | * | * | * |
| 183 | *MLL* | * | * | * |
| 184 | *MLL2* |  | * | * |
| 185 | *MLL3* |  | * | * |
| 186 | *MPL* | * | * | * |
| 187 | *MRE11A* | * | * | * |
| 188 | *MSH2* | * | * | * |
| 189 | *MSH6* | * | * | * |
| 190 | *MTF2* |  |  | * |
| 191 | *MTHFR* |  |  | * |
| 192 | *MTOR* | * | * | * |
| 193 | *MTUS2* | * | * | * |
| 194 | *MYC* | * | * | * |
| 195 | *MYCL1* | * | * | * |
| 196 | *MYCN* | * | * | * |
| 197 | *MYCNOS* | * |  |  |
| 198 | *MYD88* |  |  | * |
| 199 | *NAT2* |  |  | * |
| 200 | *NBR1* | * |  |  |
| 201 | *NBR2* | * |  |  |
| 202 | *NF1* | * | * | * |
| 203 | *NF2* | * | * | * |
| 204 | *NFE2L2* | * | * | * |
| 205 | *NKX2-1* | * | * | * |
| 206 | *NOTCH1* | * | * | * |
| 207 | *NOTCH2* | * | * | * |
| 208 | *NOTCH3* | * | * | * |
| 209 | *NOTCH4* | * | * | * |
| 210 | *NPAT* | * |  |  |
| 211 | *NPM1* | * | * | * |
| 212 | *NQO1* |  |  | * |
| 213 | *NRAS* | * | * | * |
| 214 | *NTHL1* | * |  |  |
| 215 | *NTRK1* | * | * | * |
| 216 | *NTRK2* | * | * | * |
| 217 | *NTRK3* | * | * | * |
| 218 | *PAK7* | * | * | * |
| 219 | *PAX5* | * | * | * |
| 220 | *PBRM1* | * | * | * |
| 221 | *PDGFRA* | * | * | * |
| 222 | *PDGFRB* | * | * | * |
| 223 | *PDPK1* | * | * | * |
| 224 | *PGAP3* | * |  |  |
| 225 | *PHF19* |  |  | * |
| 226 | *PHF6* | * | * | * |
| 227 | *PIK3CA* | * | * | * |
| 228 | *PIK3CD* |  |  | * |
| 229 | *PIK3R1* | * | * | * |
| 230 | *PIM1* |  | * | * |
| 231 | *PKD1* | * |  |  |
| 232 | *PLK1* |  | * | * |
| 233 | *PRKDC* |  | * | * |
| 234 | *PTCH1* | * | * | * |
| 235 | *PTEN* | * | * | * |
| 236 | *PTK2* | * | * | * |
| 237 | *PTK2B* | * | * | * |
| 238 | *PTPN11* | * | * | * |
| 239 | *PTPRD* | * | * | * |
| 240 | *PVT1* | * |  |  |
| 241 | *RAF1* | * | * | * |
| 242 | *RB1* | * | * | * |
| 243 | *RBM34* | * |  |  |
| 244 | *REL* | * | * | * |
| 245 | *RET* | * | * | * |
| 246 | *RICTOR* | * | * | * |
| 247 | *ROR2* |  |  | * |
| 248 | *ROS1* |  | * | * |
| 249 | *RPTOR* | * | * | * |
| 250 | *RRM1* |  | * | * |
| 251 | *RUNX1* | * | * | * |
| 252 | *RUNX1T1* | * | * | * |
| 253 | *SCN5A* |  |  | * |
| 254 | *SDCCAG8* | * |  |  |
| 255 | *SETD2* |  | * | * |
| 256 | *SETDB1* | * | * | * |
| 257 | *SH2D2A* | * |  |  |
| 258 | *SLCO1B1* |  |  | * |
| 259 | *SLTM* | * |  |  |
| 260 | *SMAD2* | * | * | * |
| 261 | *SMAD3* | * | * | * |
| 262 | *SMAD4* | * | * | * |
| 263 | *SMARCA4* | * | * | * |
| 264 | *SMARCB1* | * | * | * |
| 265 | *SMO* | * | * | * |
| 266 | *SMOX* | * |  |  |
| 267 | *SNAPC5* | * |  |  |
| 268 | *SOCS1* | * | * | * |
| 269 | *SPEN* | * | * | * |
| 270 | *SRC* | * | * | * |
| 271 | *STAT3* | * | * | * |
| 272 | *STK11* | * | * | * |
| 273 | *SUFU* | * | * | * |
| 274 | *SULT1A1* |  |  | * |
| 275 | *SUPT4H1* |  |  | * |
| 276 | *SUPT5H* |  |  | * |
| 277 | *TCF3* |  |  | * |
| 278 | *TCF4* | * | * | * |
| 279 | *TCF7L2* | * |  |  |
| 280 | *TERT* | * | * | * |
| 281 | *TET1* | * | * | * |
| 282 | *TET2* | * | * | * |
| 283 | *TGFBR2* | * | * | * |
| 284 | *TMPRSS2* |  |  | * |
| 285 | *TNFAIP3* | * | * | * |
| 286 | *TNK2* |  |  | * |
| 287 | *TOP1* | * | * | * |
| 288 | *TOP2A* |  | * | * |
| 289 | *TP53* | * | * | * |
| 290 | *TPMT* |  |  | * |
| 291 | *TRIM35* | * |  |  |
| 292 | *TRPV6* | * |  |  |
| 293 | *TSC1* | * | * | * |
| 294 | *TSC2* | * | * | * |
| 295 | *TSHR* | * | * | * |
| 296 | *TSPAN31* | * |  |  |
| 297 | *TYK2* |  | * | * |
| 298 | *TYMS* |  |  | * |
| 299 | *UGT1A1* |  |  | * |
| 300 | *UTY* | * | * | * |
| 301 | *VHL* | * | * | * |
| 302 | *VKORC1* |  |  | * |
| 303 | *WDR20* | * |  |  |
| 304 | *WHSC2* |  |  | * |
| 305 | *WRAP53* | * |  |  |
| 306 | *WT1* | * | * | * |
| 307 | *WT1-AS* | * |  |  |
| 308 | *ZNF646* | * |  |  |
| 309 | *ZNF668* | * | * | * |

**Supplemental Table 1.** **List of genes that were included in ClinSeq versions 4, 5 and 7 of the UNCseq**™ **project.**
