# Supplementary material for: Combined Targeted DNA Sequencing in Non-Small Cell Lung Cancer (NSCLC) Using UNCseq and NGScopy, and RNA Sequencing Using UNCqeR for the Detection of Genetic Aberrations in NSCLC
Source: PLoS One. 2015 Jun 15;10(6):e0129280. doi: 10.1371/journal.pone.0129280 (PMC4468211; doi:10.1371/journal.pone.0129280)
Supplement: S2 Table — Abbreviations: SF, snap-frozen; FFPE, formalin-fixed paraffin-embedded; bp, base pair; PCR, polymerase chain reaction; CLIA, clinical laboratory improvement amendments. (DOCX) [file pone.0129280.s006.docx]

| **Cohort** | **07-0120** | **11-1115** |
| --- | --- | --- |
| **Sequencing** |  |  |
| # of Samples (SF/FFPE) | 100/0 | 11/13 |
| Matched Germline DNA | No | Yes |
| Paired-End | No | Yes |
| Read Length (bp) | 100 | 100 |
| Library Prep. Reagent | Agilent SureSelect  (ClinSeq v4, v5) | Agilent SureSelect  (ClinSeq v7) |
| Sequencer | HiSeq 2000 | HiSeq 2000 |
| **Bioinformatics Pipeline** | 2012-2013 | Until August 2014 |
| Alignment | BWA (0.6.2) | BWA MEM (0.7.4) |
| Realignment | GATK (0.6.2) | ABRA (0.46) |
| SNV/Indel Calling | VarScan (2.3.6) | Strelka (2013) |
| **Validation** | Sanger sequencing (non-CLIA ) | Sanger/PCR (CLIA) |

**Supplemental Table 2. Key differences of the UNCseq™ sequencing, bioinformatics pipeline, and validation of tissue samples between the 07-0120 and 11-1115 cohort.** *Abbreviations*: SF, snap-frozen; FFPE, formalin-fixed paraffin-embedded; bp, base pair; PCR, polymerase chain reaction; CLIA, clinical laboratory improvement amendments.
