# Supplementary material for: Combined Targeted DNA Sequencing in Non-Small Cell Lung Cancer (NSCLC) Using UNCseq and NGScopy, and RNA Sequencing Using UNCqeR for the Detection of Genetic Aberrations in NSCLC
Source: PLoS One. 2015 Jun 15;10(6):e0129280. doi: 10.1371/journal.pone.0129280 (PMC4468211; doi:10.1371/journal.pone.0129280)
Supplement: S3 Table — Abbreviations: SF, snap-frozen; FFPE, formalin-fixed paraffin-embedded; SNV, single-nucleotide variations; CLIA, clinical laboratory improvement amendments; SOC, standard of care assays used by CLIA-certified Molecular Pathology laboratories to report mutations for clinical use; PPH2, PolyPhen 2; MAF, mutant allele frequency; Nonsyn., Nonsynonymous; Nonframe., Nonframeshift; Frame., Frameshift; ins., insertion; del.,deletion; dam., damage. (DOCX) [file pone.0129280.s007.docx]

| **Sample ID** | **Type of** |  |  | **Mutations** |  |  |  | **Validation** |  | **Control** |  |
| --- | --- | --- | --- | --- | --- | --- | --- | --- | --- | --- | --- |
|  | **Tumor Specimen** | **Genomic Position** | **Gene Mutations^1^** | **Mutation Type** | **PPH2 Level** | **PPH2 Score** | **MAF** |  | **Matched**  **Normal** | **Pooled**  **Normal** | **Normal-**  **Free** |
| **NSCLC02** | SF | *chr3:178936091* | *PIK3CA* (p.E545K) | Nonsyn. SNV | Probably dam. | 0.909 | 0.14 | CLIA | YES | YES | YES |
| **NSCLC04** | SF | No Mutations |  |  |  |  |  |  |  |  |  |
| **NSCLC05** | SF | *chr7:55242467-55242481* | *EGFR* (p.746_751del) | Nonframe. del. | N/A | N/A | N/A | SOC | YES | YES | YES |
|  |  | *chr17:7577120* | *TP53* (p.R141H) | Nonsyn. SNV | Probably dam. | 0.989 | 0.15 |  | YES | YES | YES |
|  |  | *chr17:29664869* | *NF1* (p.W2225X) | Stopgain | N/A | N/A | 0.06 |  |  | YES | YES |
| **NSCLC06** | SF | *chr4:55955880* | *KDR* (p.L1094F) | Nonsyn. SNV | Probably dam. | 1.000 | 0.36 | CLIA | YES | YES | YES |
|  |  | *chr12:25398284* | *KRAS* (p.G12V) | Nonsyn. SNV | Probably dam. | 0.978 | 0.63 | SOC | YES | YES | YES |
|  |  | *chr17:7577142* | *TP53* (p.G266X) | Stopgain | N/A | N/A | 0.35 |  | YES | YES | YES |
|  |  | *chr17:7578457* | *TP53* (p.R158L) | Nonsyn. SNV | Probably dam. | 1.000 | 0.36 |  | YES | YES | YES |
|  |  | *chr17:7579414* | *TP53* (p.W91X) | Stopgain | N/A | N/A | 0.38 |  | YES | YES | YES |
|  |  | *chr19:30313189* | *CCNE1* (p.P295S) | Nonsyn. SNV | Benign | 0.004 | 0.22 |  |  | YES | YES |
| **NSCLC11** | FFPE | No Mutations |  |  |  |  |  |  |  |  |  |
| **NSCLC15** | FFPE | No Mutations |  |  |  |  |  |  |  |  |  |
| **NSCLC17** | SF | *chr8:93004034* | *RUNX1T1* (p.R334L) | Nonsyn. SNV | Possibly dam. | 0.869 | 0.28 |  | YES | YES | YES |
|  |  | *chr9:21974677* | *CDKN2A* (p.Q50H) | Nonsyn. SNV | Probably dam. | 0.999 | 0.65 |  | YES | YES | YES |
|  |  | *chr10:8100408* | *GATA3* (p.G128W) | Nonsyn. SNV | Probably dam. | 0.942 | 0.36 |  | YES | YES | YES |
|  |  | *chr17:7578457* | *TP53* (p.R158L) | Nonsyn. SNV | Probably dam. | 1.000 | 0.72 |  | YES | YES | YES |
|  |  | *chr19:1220629* | *STK11* (p.S216F) | Nonsyn. SNV | Probably dam. | 0.999 | 0.71 |  | YES | YES | YES |
| **NSCLC18** | SF | No Mutations |  |  |  |  |  |  |  |  |  |
| **NSCLC19** | SF | *chr1:120612013* | *NOTCH2* (p.A3V) | Nonsyn. SNV | Possibly dam. | 0.739 | 0.12 |  |  | YES | YES |
| **NSCLC20** | FFPE | No Mutations |  |  |  |  |  |  |  |  |  |
| **NSCLC21** | SF | No Mutations |  |  |  |  |  |  |  |  |  |
| **NSCLC23** | FFPE | *chr7:55259515* | *EGFR* (p.L858R) | Nonsyn. SNV | Probably dam. | 0.999 | 0.32 | SOC | YES | YES | YES |
| **NSCLC25** | SF | *chr17:7577556* | *TP53* (p.C242F) | Nonsyn. SNV | Probably dam. | 1.000 | 0.07 |  | YES | YES | YES |
| **NSCLC27** | FFPE | *chr11:533925* | *HRAS* (p.V44A) | Nonsyn. SNV | Benign | 0.290 | 0.07 | CLIA | YES | YES | YES |
| **NSCLC28** | PAR | No Mutations |  |  |  |  |  |  |  |  |  |
| **NSCLC29** | PAR | *chr13:49027133* | *RB1* (p.S567fs) | Frame. del. | N/A | N/A | N/A |  | YES | YES | YES |
|  |  | *chr17:7577574* | *TP53* (p.Y236C) | Nonsyn. SNV | Probably dam. | 0.995 | 0.62 |  | YES | YES | YES |
|  |  | *chr17:37880981* | *ERBB2* (p.E770delinsEAYVM) | Nonframe. ins. | N/A | N/A | N/A | CLIA | YES | YES | YES |
| **NSCLC30** | SF | *chr12:25398284* | *KRAS* (p.G12V) | Nonsyn. SNV | Probably dam. | 0.978 | 0.49 |  | YES | YES | YES |
|  |  | *chr17:7577124* | *TP53* (p.V272M) | Nonsyn. SNV | Probably dam. | 0.998 | 0.65 |  | YES | YES | YES |
| **NSCLC31** | PAR | *chr1:120465262* | *NOTCH2* (p.V1667I) | Nonsyn. SNV | Benign | 0.001 | 0.48 |  |  | YES | YES |
|  |  | *chr9:80537112* | *GNAQ* ( p.T96S) | Nonsyn. SNV | Benign | 0.001 | 0.09 |  |  | YES | YES |
|  |  | *chr15:66777333* | *MAP2K1* (p.E233D) | Nonsyn. SNV | Probably dam. | 1.000 | 0.08 | CLIA | YES | YES | YES |
|  |  | *chr17:7577114* | *TP53* (p.C275F) | Nonsyn. SNV | Probably dam. | 1.000 | 0.19 |  | YES | YES | YES |
| **NSCLC33** | FFPE | *chr2:178098944* | *NFE2L2* (p.R34Q) | Nonsyn. SNV | Probably dam. | 0.998 | 0.55 |  | YES | YES | YES |
|  |  | *chr17:7578395* | *TP53* (p.H179N) | Nonsyn. SNV | Probably dam. | 1.000 | 0.60 |  | YES | YES | YES |
| **NSCLC35** | FFPE | *chr9:80537112* | *GNAQ* ( p.T96S) | Nonsyn. SNV | Benign | 0.001 | 0.08 |  |  | YES | YES |
|  |  | *chr10:43595990* | *RET* (p.V53F) | Nonsyn. SNV | Probably dam. | 0.991 | 0.35 | CLIA | YES | YES | YES |
|  |  | *chr17:7578395* | *TP53* (p.H179D) | Nonsyn. SNV | Probably dam. | 1.000 | 0.37 |  | YES | YES | YES |
| **NSCLC37** | FFPE | *chr7:116411990* | *MET* (p.T1010I) | Nonsyn. SNV | Probably dam. | 0.999 | 0.46 |  |  | YES | YES |
|  |  | *chr9:80537112* | *GNAQ* ( p.T96S) | Nonsyn. SNV | Benign | 0.001 | 0.08 |  |  | YES | YES |
|  |  | *chr10:123243222* | *FGFR2* (p.T765K) | Nonsyn. SNV | Benign | 0.154 | 0.16 | CLIA | YES | YES | YES |
|  |  | *chr17:7579358* | *TP53* (p.R110P) | Nonsyn. SNV | Probably dam. | 0.953 | 0.15 |  | YES | YES | YES |
| **NSCLC38** | FFPE | *chr7:116339642* | *MET* (p.E168D) | Nonsyn. SNV | Possibly dam. | 0.794 | 0.50 |  |  | YES | YES |
|  |  | *chr7:116436011* | *MET* (p.R1354W) | Nonsyn. SNV | Probably dam. | 0.922 | 0.10 | CLIA | YES | YES | YES |
|  |  | *chr17:37880997* | *ERBB2* (p.G776delinsVC) | Nonframe. ins. | N/A | N/A | N/A | CLIA | YES | YES | YES |
| **NSCLC40** | FFPE | *chr2:30143134* | *ALK* (p.S131F) | Nonsyn. SNV | Benign | 0.015 | 0.18 | CLIA | YES | YES | YES |
|  |  | *chr4:153244147* | *FBXW7* (p.G670fs) | Frame. ins. | N/A | N/A | N/A | SOC | YES | YES | YES |
|  |  | *chr9:80537112* | *GNAQ* ( p.T96S) | Nonsyn. SNV | Benign | 0.001 | 0.06 |  |  | YES | YES |
|  |  | *chr12:25398285* | *KRAS* (p.G12C) | Nonsyn. SNV | Probably dam. | 0.993 | 0.21 | SOC | YES | YES | YES |
|  |  | *chr17:7577096* | *TP53* (p.D281G) | Nonsyn. SNV | Probably dam. | 1.000 | 0.25 | SOC | YES | YES | YES |
| **NSCLC46** | SF | *chr5:112175240* | *APC* (p.E1317Q) | Nonsyn. SNV | Benign | 0.003 | 0.47 |  |  | YES | YES |

**Supplemental Table 3. List of mutations identified in the 24 tumor tissue samples collected as part of the 11-1115 tissue cohort using three methods to call mutations (matched normal, pooled normal, and normal free variant calling).** Abbreviations: SF, snap-frozen; FFPE, formalin-fixed paraffin-embedded; SNV, single-nucleotide variations; CLIA, clinical laboratory improvement amendments; SOC, standard of care assays used by CLIA-certified Molecular Pathology laboratories to report mutations for clinical use; PPH2, PolyPhen 2; MAF, mutant allele frequency; Nonsyn., Nonsynonymous; Nonframe., Nonframeshift; Frame., Frameshift; ins., insertion; del.,deletion; dam., damage.
