# Supplementary material for: Combined Targeted DNA Sequencing in Non-Small Cell Lung Cancer (NSCLC) Using UNCseq and NGScopy, and RNA Sequencing Using UNCqeR for the Detection of Genetic Aberrations in NSCLC
Source: PLoS One. 2015 Jun 15;10(6):e0129280. doi: 10.1371/journal.pone.0129280 (PMC4468211; doi:10.1371/journal.pone.0129280)
Supplement: S1 Text — (DOCX) [file pone.0129280.s008.docx]

**Supplemental Text**

A. *Depth of coverage (Cd), or read depth (RD)* is defined as the total number ( $N_{i}$ ) of reads overlapping each and every position $i$ in the target genomic regions of query divided by the total length ($L_{target}$) of these target genomic regions as shown in the following formula:

$$C_{d}=\frac{\sum_{i=1}^{L} N_{i}}{L_{target}}$$

Depth of coverage reflects the average times a given region has been sequenced by independent reads.

B. *Breadth of coverage (Cb)*, or capture sensitivity, is defined as the proportion of the target genomic regions ($L_{sequenced}$) that have been covered/sequenced relative to the total length ($L_{target}$) of these target genomic regions, as shown in the following formula:

$$C_{b}=\frac{L_{sequenced}}{L_{target}}$$

Usually, a given NGS dataset will not encompass the entirety of the target genomic regions, because certain regions are difficult to sequence and/or map. Therefore, breadth of coverage reflects how broad the target genomic regions have been covered.

C. *On-target rate*, or capture specificity, is defined as the proportion of mapped bases ($N_{target}$) that fall into the target genomic regions relative to the number of overall mapped bases ( $N_{total}$ ), as shown in the following formula:

$$R_{on}=\frac{N_{target}}{N_{total}}$$

D. *Expected mutant allele count* ($EMAC, or E$) by RNA-seq, is defined as follow:

$$E={Depth}_{RNA} \times{MAF}_{DNA}$$

, where ${Depth}_{RNA}$ is the coverage depth by RNA-seq and ${MAF}_{DNA}$ is the observed mutant allele frequency (MAF) by DNA-seq.
